# Single-base resolution maps of cultivated and wild rice methylomes and regulatory roles of DNA methylation in plant gene expression

**DOI:** 10.1186/1471-2164-13-300

**Published:** 2012-07-02

**Authors:** Xin Li, Jingde Zhu, Fengyi Hu, Song Ge, Mingzhi Ye, Hui Xiang, Guojie Zhang, Xiaoming Zheng, Hongyu Zhang, Shilai Zhang, Qiong Li, Ruibang Luo, Chang Yu, Jian Yu, Jingfeng Sun, Xiaoyu Zou, Xiaofeng Cao, Xianfa Xie, Jun Wang, Wen Wang

**Affiliations:** 1 CAS-Max Planck Junior Research Group, State Key Laboratory of Genetic Resources and Evolution, Kunming Institute of Zoology, Chinese Academy of Sciences, Kunming, 650223, China; 2 BGI-Shenzhen, Shenzhen, 518083, China; 3 Shanghai Cancer Institute, Renji Hospital affiliated to School of Medicine, Shanghai Jiaotong University, LN 2200/25, Xietu Road, Shanghai, 200032, China; 4 Center for Basic and Translational Epigenetic Research of Diseases, School of Life Science, Anhui Medical University, Hefei, 230032, China; 5 Food Crops Research Institute, Yunnan Academy of Agricultural Sciences, Kunming, 650205, China; 6 State Key Laboratory of Systematic and Evolutionary Botany, Institute of Botany, Chinese Academy of Sciences (CAS), Beijing, 100093, China; 7 School of Bioscience and Biotechnology, South China University of Technology, Guangzhou, 510641, China; 8 The State Key Laboratory of Plant Genomics, National Center for Plant Gene Research, Institute of Genetics and Developmental Biology, Chinese Academy of Sciences, Beijing, 100101, China; 9 Department of Biology, Virginia State University, Petersburg, VA, 23806, USA; 10 Department of Biology, University of Copenhagen, Copenhagen, DK-2200, Denmark

**Keywords:** Cultivated and wild rice, Methylomes, Transcriptional termination regions (TTRs), Gene expression

## Abstract

**Background:**

DNA methylation plays important biological roles in plants and animals. To examine the rice genomic methylation landscape and assess its functional significance, we generated single-base resolution DNA methylome maps for Asian cultivated rice *Oryza sativa* ssp*. japonica*, *indica* and their wild relatives, *Oryza rufipogon* and *Oryza nivara*.

**Results:**

The overall methylation level of rice genomes is four times higher than that of *Arabidopsis*. Consistent with the results reported for *Arabidopsis*, methylation in promoters represses gene expression while gene-body methylation generally appears to be positively associated with gene expression**.** Interestingly, we discovered that methylation in gene transcriptional termination regions (TTRs) can significantly repress gene expression, and the effect is even stronger than that of promoter methylation. Through integrated analysis of genomic, DNA methylomic and transcriptomic differences between cultivated and wild rice, we found that primary DNA sequence divergence is the major determinant of methylational differences at the whole genome level, but DNA methylational difference alone can only account for limited gene expression variation between the cultivated and wild rice. Furthermore, we identified a number of genes with significant difference in methylation level between the wild and cultivated rice.

**Conclusions:**

The single-base resolution methylomes of rice obtained in this study have not only broadened our understanding of the mechanism and function of DNA methylation in plant genomes, but also provided valuable data for future studies of rice epigenetics and the epigenetic differentiation between wild and cultivated rice.

## Background

DNA methylation is an epigenetic modification mechanism that plays essential roles in diverse biological processes [1]. It has also been proposed to be an alternative inheritance system playing an important role in evolution [2,3], as many case studies in plants and animals have revealed that differentially methylated alleles could create heritable phenotypic changes across generations [4-10], including some agronomically important traits in rice [11]. Recently, single-base resolution methylome maps of a dicot plant (*Arabidopsis thaliana*), human and silkworm have been successfully generated by whole-genome sequencing bisulfite-treated genomic DNA using next-generation sequencing technology (BS-Seq), which revealed more elaborate patterns and functional effects of DNA methylation at the whole-genome level [12-15].

Rice is not only one of the most important crops as the primary food source for more than half of the world’s population, but also an important model system for the evolutionary study of cereals and the molecular study of monocot plants. DNA methylation serves various important functions and thus has been of great interest to rice geneticists and breeders. Pioneer studies of epigenetic modifications in rice, including DNA and histone methylation, using traditional methylated DNA enrichment method suggested possible functional roles of DNA methylation in rice [16,17], but this approach is difficult to discriminate major genomic elements including promoters, gene bodies, transposons, and repeats. Recently two other studies comparing methylation patterns among many species using BS-Seq technology briefly reported genome methylation patterns for *japonica* rice strain *Nipponbare*[18,19]. However, the two *Nipponbare* methylomes had relatively low sequencing coverage (< 4× per base for each strand). Furthermore, one of the studies [18] used different tissues to obtain methylomic (from leaves) and transcriptomic (from shoots) profiles and the other did not obtain gene expression data at all, which made it difficult to accurately analyze the regulatory effects of DNA methylation in rice. As acknowledged by Feng et al. [19], such a low genome-wide sequencing depth permitted good assessment of the level of methylation of major genomic elements including genes, transposons, and repeats, but was not sufficient for quantifing the methylation level at individual cytosines. Furthermore, to what extent and how the cultivated rice has evolved divergent DNA methylation pattern from its wild relative species still need to be addressed.

In this study, we generated single-base-resolution DNA methylomic maps as well as transcriptomic profiles for young panicles of the two Asian cultivated rice subspecies, *Oryza sativa* ssp*. japonica* and *O. sativa* ssp*. indica*, and their wild relatives, *Oryza rufipogon* and *Oryza nivara*. The panicle is an important organ showing strong differentiation between cultivated and wild rice and directly affects the major yield components including the number of spikelets and the percentage of filled grains [20]. The high-resolution DNA methylomes of cultivated and domestic rice will not only serve as references for future molecular studies of rice epigenetics but also shed new lights into epigenetic mechanisms of plant domestication.

## Results and discussions

### Methylation landscapes in rice

To investigate the general methylation patterns of rice as well as the DNA methylation divergence between cultivated and wild rice, we included in our samples both subspecies of Asian cultivated rice, *Oryza sativa* spp. *japonica* (represented by Dianjingyou1, bred by the Yunnan Academy of Agricultural Sciences, China, which is a typical *japonica* rice type mainly suitable to be planted in Yunnan) and *indica* (IR64, from the International Rice Research Institute, IRRI), and their wild relatives, *Oryza rufipogon* (Accession 105327, originally collected from Sri Lanka and provided by IRRI) and *Oryza nivara* (Accession 105426, originally collected from India and provided by IRRI).

For each of the four rice lines, a young panicle was used to generate a methylomic map with the BS-Seq method. 170–320 million sequencing reads were generated for each of the four samples, respectively. After removing low-quality and clonal reads (artificially generated during amplification of bisulfate-treated DNA in constructing sequencing libraries, see Material and methods for details), 58–176 million uniquely mapped high-quality reads were retained for each of the four rice lines, yielding 2.6-9.2 gigabases (Gb) of data which cover 76-91% of the reference Nipponbare genome (International Rice Genome Sequencing Project, 2005), respectively (Table 1). The read depths range from 4.5× to 13.5× per base for each DNA strand (Table 1). Because *japonica* has the best reference genome and we obtained the highest genome coverage and sequencing depth for the *japonica* strain Dianjingyou1, here we use the *japonica* accession as the representative to describe the general methylation landscape of rice, with the differences between wild and cultivated rice being discussed later.

**Table 1:** Data description of BS-Seq reads for the four rice samples

**Sample**	***Japonica***	***Indica***	***O. rufipogon***	***O. nivara***
**Raw reads number/data production (Gb)**	320,730,854/16.2	170,784,798/7.5	171,346,432/7.5	203,796,688/9.0
**Effective reads number/data production (Gb)**	175,749,760/9.2	58,423,521/2.6	68,773,618/3.0	81,784,095/3.6
**Genome coverage**	91%	76%	78%	77%
**Average read depth per base per strand**	13.5 ×	4.54 ×	5.17 ×	6.25×

We used the unmethylated chloroplast genome [21] to calculate the sum of non-conversion rate and T-C sequencing error rate, which is as low as 0.47% to 1.17% for the four samples respectively (Table 2). Using these values we then conducted binomial tests with false positive rate below 5% to exclude those mCs that may be the results of non-conversion of cytosines during our bisulfite treatment or T to C sequencing errors during the base calling process. After this correction, we identified 35,598,491 methylated cytosines (mCs) accounting for 24.3% of all covered cytosines throughout the reference genome in the *japonica* Dianjingyou1 (Table 2), which is four times higher than that of *Arabidopsis*[13] in terms of mC density. The percentages of mCs in CG, CHG (with H being A, C or T) and CHH contexts are 54.7%, 37.3% and 12.0%, respectively while the average methylation level in the three contexts are 44.5%, 24.1% and 4.7%, respectively, with methylation level being defined as the proportion of reads showing mC among all reads covering the same cytosine site. Both of these measures reveal that rice has much higher level of genome-wide DNA methylation than *A. thaliana*[12,13] (Table 2). These patterns are more or less the same in *indica* and the two wild rice species (Table 2), and are consistent with the two previous studies of rice leaf methylomes [18,19].

**Table 2:** Conversion rate and methylation pattern for the four rice samples

**Sample**	**Japonica**			**Indica**		
error rate^*^	**1.12%**					
**Methylation**	Methylcytosine number	Methylation density	Average methylation level of all cytosines	Methylcytosine number	Methylation density	Average methylation level of all cytosines
**Total**	35,598,491	24.27%	15.40%	22,559,747	18.85%	9.81%
**CG**	14,989,765	54.68%	44.46%	9,881,382	45.17%	27.77%
**CHG**	9,238,307	37.31%	20.14%	5,553,755	27.29%	13.48%
**CHH**	11,370,419	12.03%	4.02%	7,124,610	9.20%	3.16%
**Sample**	***O. rufipogon***			***O. nivara***		
**error rate**	0.47%			0.94%		
**Methylation**	Methylcytosine number	Methylation density	Average methylation level of all cytosines	Methylcytosine number	Methylation density	Average methylation level of all cytosines
**Total**	23,358,199	18.95%	9.05%	24,308,799	20.75%	13.79%
**CG**	10,333,979	46.75%	30.82%	9,991,032	49.63%	45.46%
**CHG**	5,785,271	27.89%	12.74%	5,735,875	29.46%	19.18%
**CHH**	7,238,949	9.00%	2.75%	8,581,892	11.07%	5.75%
**Sample**	***Arabidopsis***[12,13]					
**error rate**						
**Methylation**	Methylation density	Average methylation level of all cytosines				
**Total**	5.26%	NA				
**CG**	NA	24.00%				
**CHG**	NA	6.70%				
**CHH**	NA	1.70%				

* The sum of non-conversion rate and T-C sequencing error rate.

**Figure 1:**
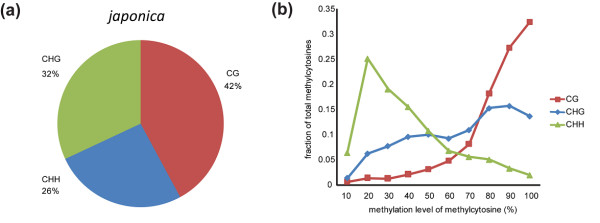
**DNA methylation pattern in the *japonica* rice (Dianjingyou1). (a)** Relative proportions of mCs in three sequence contexts. **(b)** Distribution of methylation level of mCs in each sequence context. Only mCs covered by at least 5 reads were used to calculate methylation level. Methylation level on the x-axis was defined as the percentage of reads showing methylated cytosine at a reference cytosine site. The y-axis indicates the fraction of total mCs calculated within bins of 10%.

**Figure 2:**
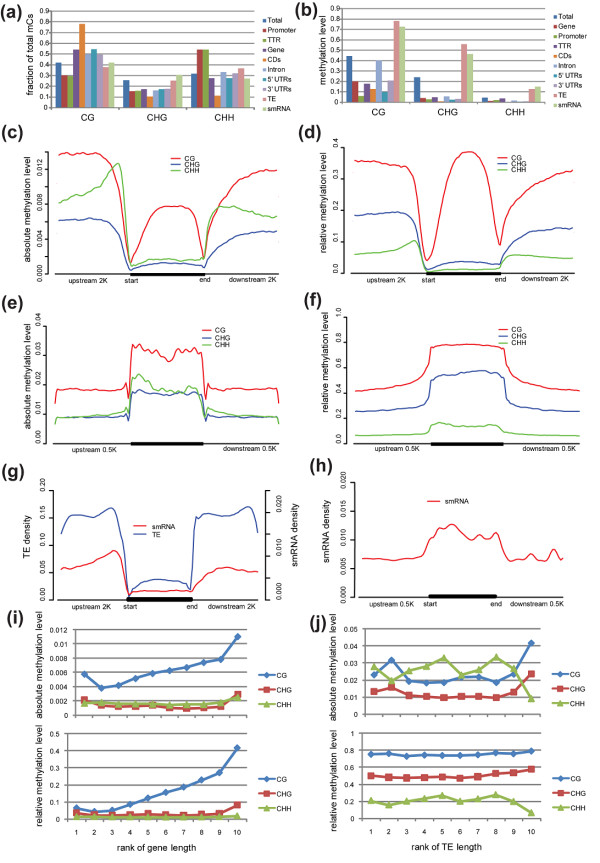
**DNA methylation patterns in different genomic regions.** Methylation patterns were characterized in following functional regions: TEs, small RNA (smRNA) loci, and genic regions including the promoter (200 bp upstream of the transcriptional start site, TSS), gene body (the entire transcribed region), and the transcriptional termination region (TTR, 200 bp downstream of transcriptional termination site). Gene body is further divided into untranslated regions (UTRs), coding regions (CDs), and introns. Methylation level, TE density and smRNA locus density were calculated across TE, gene body and their flanking sequences using an overlapping sliding window of 5% of the sequence length at a step of 2.5% of the sequence length. **(a)** Fraction of total mCs in each sequence context for different genomic regions. **(b)** Relative methylation level (total methylation level of mCs divided by sequence length of the calculated region) in each sequence context for different genomic regions. Distributions of absolute methylation level (total methylation level of mCs divided by total number of cytosine sites in the calculated region) **(c)** and relative methylation level **(d)** in gene body and 2-kb flanking sequences on both sides. Absolute **(e)** and relative **(f)** methylation level distributions in TE and 0.5-kb flanking sequences on both sides. **(g)** TE and smRNA density distributions in gene body and 2-kb flanking sequences. **(h)** smRNA density distribution throughout TE and 0.5 kb-flanking sequences. Relationships between methylation level and sequence length in genes **(i)** and TE regions **(j)**, in which both absolute (top) and relative (bottom) methylation levels were analyzed.

The genome-wide methylation patterns with respect to the genomic structure in rice young panicles are similar to those observed in *A. thaliana*[12,13,22] and in rice leaves [18,19], particularly in the relative prevalence of mCs in the contexts of GG, CHG, and CHH (Figure 1a), the tendency toward hypermethylation in CG context but hypomethylation in CHH (Figure 1b), the high methylation level in transposable elements (TEs) and the relatively low level of methylation in genic regions, the enrichment of CG methylation in gene bodies (Figure 2a-f), and the enrichment of small RNA loci in TEs and depletion in genic regions (Figure 2g and 2h).

### Difference of methylation landscapes between rice and *A. thaliana*

**Figure 3:**
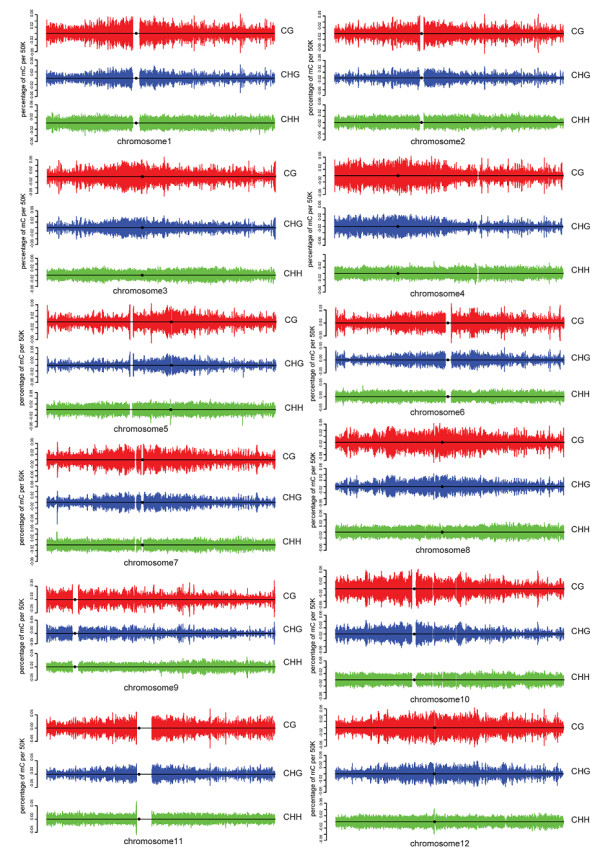
**Distribution of mCs on the sense and antisense strands of rice chromosomes for each sequence context.** The sliding window size is 50 kb and the step size is 25 kb. The black circle indicates the centromeric position of a chromosome. Some centromeric regions of chromosomes have not been completely sequenced and thus are displayed as gaps in the figure.

However, in contrast to the significantly enriched methylation in all the three sequence contexts around the centromeric regions in *A. thaliana* genome [13], rice shows much less difference between centromeric and non-centromeric regions with only slight CG and CHG methylation enrichment around the centromeric regions and almost uniform distribution of CHH mCs across entire chromosomes (Figure 3). This is probably due to the fact that compared to *A. thaliana*, rice has significantly larger amount of pericentromeric heterochromatin [23,24] and much higher proportion of TEs distributed across the genome, both of which are heavily methylated.

In addition, we found a positive correlation between sequence length and methylation density for genes (Figure 2i), but not for TEs (Figure 2j), which is different from the case in *A. thaliana* wherein both genes and TEs showed positive correlation between sequence length and methylation [12]. This might be due to the fact that TEs in rice genome are almost saturated with mCs regardless of their length. All the above results are consistently found in all the four rice samples (see Additional files 1 and 2 for results of other samples), showing these are general patterns in both cultivated and wild rice. These differences between rice and *Arabidopsis* suggest that differences in genomic organization and TE composition among plants could result in different epigenomic landscapes.

### Regulatory roles of promoter and gene-body DNA methylation in gene expression

To assess the effects of rice DNA methylation on gene expression, we also generated genome-wide gene expression profiles for all the four samples (Table 3) using Digital Gene Expression tag profiling (DGE) technology, which combines the classic SAGE (Serial Analysis of Gene Expression) with the Illumina sequencing techniques. In total we obtained 7,343,629 to 7,774,577 raw reads with 599,304 to 648,947 unique tag sequences for each of the four samples. After filtration and mapping reads to 24,955 non-redundant full-length cDNAs (FL-cDNAs) of rice, our DGE data cover 79% to 83% of all FL-cDNAs with a one-tag-one-cDNA relationship (Table 3).

**Table 3:** DGE data description for the four rice samples

**Sample**	***Japonica***	***Indica***	***O. rufipogon***	***O. nivara***
**Raw/distinct tag number**	7,662,276/648,947	7,418,013/562,392	7,774,577/561,691	7,343,629/599,304
**Total/distinct tag number used in analysis**	7,589,226/612,176	7,413,872/558,498	7,769,442/556,909	7,334,416/591,759
**Genes with CATG sites**	24,611 (98.62%)	24,611 (98.62%)	24,611 (98.62%)	24,611 (98.62%)
**Tags with perfect match to unique gene**	4,729,194	4,295,776	4,579,715	4,162,572
**Genes with perfect match to unique tags**	20,682 (82.88%)	19,804 (79.36%)	20,095 (80.52%)	19,745 (79.12%)

Although some previous studies have also explored the relationship between DNA methylation and gene expression in rice [16,18], their low resolution of genome-wide methylated cytokines (mCs) [16] or utilization of different tissues in generating transcriptomic and methylomic profiles [18] created the need for more elaborate methylomic studies to comprehensively unveil high resolution rice methylomes and detailed functional effects of rice DNA methylation. Here we shall mainly use the *japonica* data to present the detailed patterns in rice, which are similar in all four samples (see Additional file 3 for results of the other three samples).

**Figure 4:**
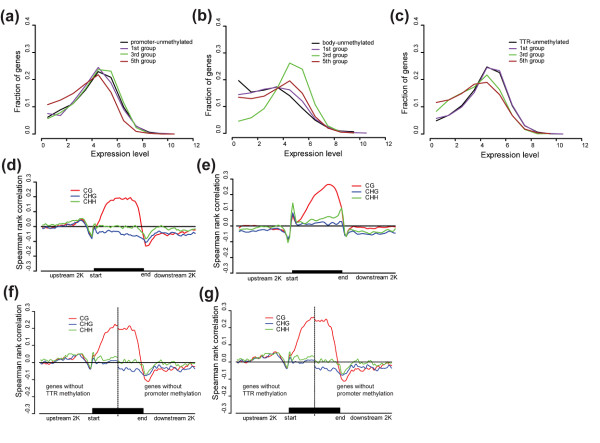
**Relationship between methylation level and gene expression in different genic regions. (a)** Promoter; **(b)** Gene body; **(c)** TTR. Methylation level was measured using the absolute methylation level but similar results were also obtained using the relative methylation level. Genes are categorized into unmethylated (black line) and methylated ones, the latter of which were further divided into five groups based on the absolute methylation level (from Group 1 of the 20% of genes with the lowest methylation level to Group 5 of the 20% with the highest methylation level). For clarity, we only display 1st, 3rd and 5th groups. Methylation-expression Spearman correlation coefficients along genes and their 2 kb-flanking regions in rice **(d)** and *Arabidopsis***(e)**. The methylation-expression Spearman correlation coefficients were also calculated in TTR and promoter regions using genes without TTR methylation (left to the dashed line) and genes without promoter methylation (right to the dashed line) respectively. Methylation was measured using absolute methylation level **(f)** or relative methylation level **(g)**. The correlation coefficients were calculated using an overlapping sliding window of 5% of sequence length at a step of 2.5% of sequence length.

Our results show that promoter-unmethylated genes have significantly higher expression level than promoter-methylated genes (*p* = 1.915e-08, Wilcoxon rank sum test), indicating that promoter methylation represses gene expression. Consistent with this conclusion, the repression effect is weak for slightly and moderately promoter-methylated genes while very strong for heavily methylated ones (Figure 4a, and see Additional file 4 for each sequence context). These results from the high resolution genome-wide data proved false the previous speculation that promoter methylation may not affect rice gene transcription [16] but are consistent with the findings from *A. thaliana*[25], human [26], and the recent rice methylome study [18], confirming that promoter methylation is a general mechanism suppressing gene expression in eukaryotes.

In contrast to promoter methylation, gene-body methylation generally appears to be positively correlated to gene expression as body-methylated genes have significantly higher expression level than body-unmethylated genes (*p* < 2.2e-16, Wilcoxon rank sum test). However, further analysis revealed complicated relationships between gene-body methylation and gene expression. At first gene expression levels increase with methylation levels, but after a certain point, heavy gene-body methylation appears to repress gene expression; and consequently genes with moderate levels of body methylation tend to have the highest expression levels (Figure 4b, see Additional file 4 for each sequence context). These observations are consistent with previous studies in *A. thaliana*[25,27] and in rice [18]. It has been proposed that gene-body methylation can prevent transcriptional initiation from cryptic sites within genes but at the cost of impeding transcriptional elongation [27]. This trade-off may have led to the observation that moderately body-methylated genes have the highest level of expression.

### Methylation in transcriptional termination region (TTR) can significantly repress gene expression

Most interestingly, our study revealed that methylation in the transcriptional termination region (TTR) is also highly correlated with gene expression. In a pattern similar to promoter methylation, the TTR-unmethylated genes are expressed at significantly higher level than TTR-methylated genes (*p* < 2.2e-16, Wilcoxon rank sum test). Moreover, an approximately monotonic negative correlation exists between TTR methylation and gene expression (Figure 4c, see Additional file 4 for each sequence context). Surprisingly, the correlation coefficient is even higher than that of promoters, especially for CG methylation (Figure 4d), suggesting that TTR methylation may play an even more important role in gene expression regulation than promoter methylation. To exclude the possibility that the negative correlation between TTR methylation and gene expression is an indirect effect caused by promoter methylation if promoter-methylated genes are also prone to have methylated TTRs, we repeated the correlation analysis for both TTR and promoter regions using genes without promoter methylation and those without TTR methylation respectively. The strong negative correlation between TTR methylation and gene expression and the higher correlation coefficients than that for promoter methylation could still be observed (Figure 4f and 4 g).

DNA methylation, together with associated histone modification, has been suggested to influence the binding of RNA polymerase to DNA and thus affect the initiation, elongation and termination of the gene transcription process [27]. It has been established that promoter methylation can repress gene expression and promoter hypomethylation may be required for genes to express efficiently [1,25]. Gene-body methylation has also been proposed to inhibit transcriptional noise in actively transcribed genes and consequently body-methylated genes usually show moderate to high level of expression [22,27,28]. It is plausible that TTR methylation could have significant effect on gene expression through interfering with transcriptional termination. Consistent with this hypothesis, active DNA demethylation mediated by the DEMETER (DME) family has been found primarily at both the 5’- and 3’- ends of genes in *A. thaliana*, suggesting a functional role of methylation in both regions [29,30].

To further investigate whether such a regulatory mechanism is shared by plants, we also examined the effects of TTR methylation on gene expression in *Arabidopsis* using existing data [13]. Consistent with the results from rice, our analysis revealed significant negative correlation between TTR methylation and gene expression in *Arabidopsis* (Figure 4e), suggesting a general regulatory role of TTR methylation both in monocot and dicot plants.

In addition, we found that the 5’-end of gene coding region is another important regulatory region showing significant positive correlation between its methylation and gene expression in *Arabidopsis*, but not in rice (Figure 4d and 4e), consistent with the *Arabidopsis*-specific CHH methylation enrichment in this region. Whether this is a dicot-specific regulation mechanism needs further studies using more plant species of both monocots and dicots. It is worth noting that the positive correlation between the 5’-end gene coding region methylation and gene expression in *Arabidopsis* could be revealed based on both the absolute (total methylation level of mCs divided by sequence length) and the relative (total methylation level of mCs divided by total number of cytosine sites in a region) methylation levels, but the correspondent CHH methylation enrichment in *Arabidopsis* could only be revealed using the absolute methylation level (Additional file 5). This may explain the failure of previous studies on *Arabidopsis* to reveal the positive effect of 5’-end gene methylation on gene expression and suggests absolute methylation level may be better than relative methylation level to affect gene expression.

### Methylome comparison between wild and cultivated rice

**Figure 5:**
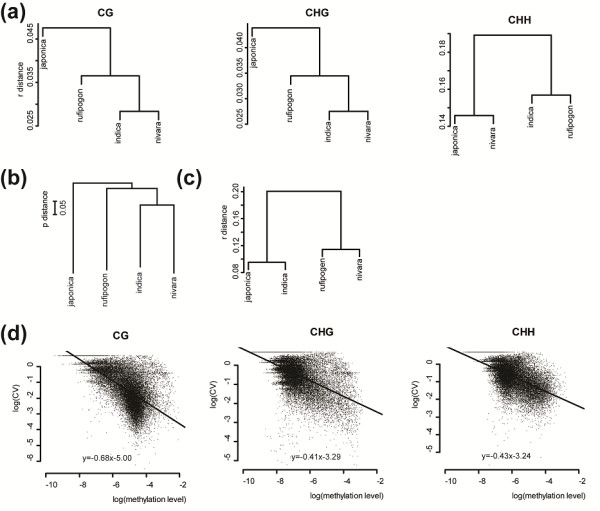
**Cluster analyses based on genome-wide cytosine methylation, SNPs and gene expression profiles of the four rice samples. (a)** Methylation tree for each sequence context. **(b)** Genomic tree based on SNPs. **(c)** Expression tree. Methylation and expression trees were constructed using the distances of correlation coefficients (*r*) of whole-genome methylation and expression profiles, respectively, among the samples. (**d**) Relationship between methylation level and their variation among species. The x-axis indicates the log_2_ transformed methylation level of each sliding-window, and y-axis indicates the log_2_ transformed CV of corresponding region among four samples.

**Figure 6:**
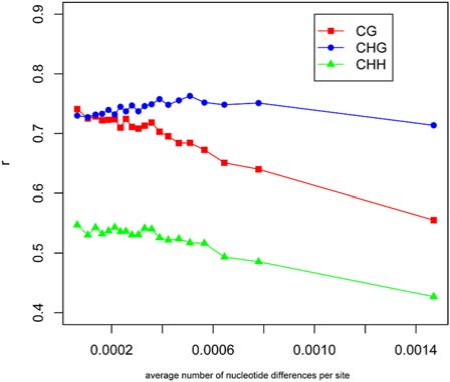
**Relationship between genetic and methylation divergence in each sequence contexts.** The average number of nucleotide differences per site (indicated by x-axis) and average Spearman correlation coefficients (*r*) of methylation level of all cytosines among samples (indicated by y-axis) were calculated for each 50-kb sliding window with a step of 25 kb across the whole genome. Then all sliding windows were classified into 20 groups with equal numbers (657 sliding windows) according to their *π* values from the lowest to the highest. The average number of nucleotide differences per site and *r* of each group were calculated and their values were plotted. Because most sliding windows have relatively small values of average number of nucleotide differences per site, the data points in the figure were enriched in the left.

To examine the divergence between cultivated and wild rice at genetic, methylation and gene expression levels, we first constructed trees based on genomic, methylomic, and gene expression data, respectively (see Materials and methods) (Figure 5a-c). The genomic DNA tree shows the same topology with the methylation-based trees except for the one constructed using CHH mCs. The consistency between the genomic and methylomic trees suggests that genetic divergence at DNA sequence level may be the major determinant of methylation patterns in the genome. We further examined the relationship between genetic and methylation divergence among samples using sliding windows across the whole genome. The average number of nucleotide differences per site and the average Spearman correlation coefficient of mCs were used to measure genetic and methylation divergence among samples, respectively (see Materials and methods). Through sliding window analysis, we found that regions with high genetic sequence divergence also have high CG methylation divergence (low correlation coefficient) among samples (Figure 6). However, this pattern was not obvious for non-CG methylation, especially CHG methylation. Taken together, these results suggested that genetic divergence may be the major determinant of CG methylation patterns which usually showed high methylation levels, while non-CG methylation level is usually low and may be easily affected by other internal/external factors besides the DNA sequence.

However, the topology of the gene expression tree is different from both genomic DNA and methylation-based trees, with the two cultivated rice subspecies tightly clustering together and the two wild rice species being most similar to each other, a pattern consistent with the phenotypic relationships among the four samples. The genome-wide gene expression divergence of panicles among the four rice subspecies/species obviously departs from the expectation under the neutral evolution model that posits that gene expression differentiation positively correlates with species’ genetic divergence [31]. Instead, it suggests rice domestication may have occurred through changes in a limited number of genes that have pleiotropic and/or cascading effects on gene expression at the whole-genome level. With considerable gene expressional and phenotypic divergence between wild and cultivated rice, the panicle might have been an major target for artificial selection during domestication, a hypothesis consistent with the finding that many yield-related traits are associated with panicles [20].

The above results, i.e., the CHH methylation tree’s inconsistency with the genomic tree and the low correlation between non-CG methylation divergence and genetic divergence, imply that high-level methylation in CG context might be more conserved than low-level methylation in CHG and CHH contexts among species. To further test this hypothesis, we calculated the coefficient of variation (CV) using a sliding window of the methylation level for each of the CG, CHG and CHH context to measure their conservation levels among species. Our results show that CG methylation has the lowest variation (8.6%), followed by CHG (11.0%) and CHH (13.7%), consistent with the decrease of their methylation level in the same order. In addition, we calculated the correlation coefficient between methylation level and its CV among four samples in gene regions for each sequence context. We found that gene methylation level showed significant negative correlation with its CV regardless of sequence contexts (Figure 5d). These results suggested that high-level methylation states are more stable during evolution.

To examine the methylation variation among species in different functional elements, we calculated the CVs of methylation level for mCs located in gene, promoter, TTR and TE regions (Additional file 6). We found that TEs’ methylation status is most conserved among species with the smallest CV, followed by genes, promoters and TTRs, consistent with the previous observation in *Arabidopsis*[32]. The methylation conservation levels of different functional elements also follow their methylation levels, again suggesting that high-level methylation states are more stable among species. Further, we compared the methylation variations among different location and sequence contexts in the same methylation level groups. We found that mCs with similar methylation levels have similar variations regardless of their genomic location or sequence contexts (Additional file 7), demonstrating that methylation level is a consistent indicator of methylation variation among species.

### Identification of differentially-methylated genes between cultivated rice and wild rice

To identify the DNA methylation changes that may be associated with rice domestication, we identified in cultivated rice 14/24/49 methylation-upregulated and 21/10/46 methylation-downregulated genes in promoter, TTR and gene body regions respectively, leading to a total of 155 non-redundant differentially-methylated genes between cultivated and wild rice (Additional file 8). We also picked 6 promoter or TTR regions in total to validate such methylation differentiation between cultivated and wild rice using the traditional bisulfite sequencing method for single genes, and the results conform to those from our whole genome BS-Seq analyses (Additional files 9,10,11,12,13,and 14), indicating the reliability of our BS-Seq results. Among the 155 genes, 11 (7.1%) show methylation-correlated 2-fold gene expression changes, but the proportion of genes with such expressional changes among differentially methylated genes is not significantly different from the proportion of all genes showing 2-fold gene expression changes between cultivated and wild rice regardless whether there is DNA methylation difference. Interestingly, a similar conclusion has also been drawn among natural accessions of *Arabidopsis thaliana* in which only 6% of differentially methylated genes have significantly different expression levels between ecotypes, and the proportion of expression-altered genes is the same as that among all genes [32]. The results from both rice and *Arabidopsis* suggest that a variety of mechanisms, including genetic changes in genes’ *cis*- or *trans-*regulators and chromatin modification, together with DNA methylation, regulate gene expression at the genomic level. However, it is also possible that subtle (less than two-fold) changes in gene expression caused by DNA methylation differences could have important consequences, particularly if those genes are of major or multiple effects. In the case of rice domestication, therefore, it still cannot be ruled out that a few key genes’ epigenetic and correlated expressional changes might have played important roles in the production of some important agronomic traits in cultivated rice [11]. Given the small sample size in this study, methylomic and transcriptomic analysis of more representative wild and cultivated rice is needed to further clarify this important issue.

## Conclusions

The high resolution DNA methylation maps for the two cultivated rice subspecies and their wild ancestors obtained in this study and the integrated analysis of genomic, epigenomic, and transcriptomic data have not only broadened our understanding of the mechanisms of gene regulation and the complicated relationships between DNA divergence, DNA methylation, and gene expression variation in plant genomes, but also provided valuable data for future studies on rice epigenetics as well as on epigenetic differentiation between wild and cultivated rice.

## Methods

### BS-Seq libraries construction and sequencing

To make our methylomes from four samples comparable, we carefully collected all samples at the same developmental stage of panicle initiation to booting. This stage is an important time point, at which rice start transitioning from vegetative growth to reproductive growth.

A single young panicle from each of the cultivated rice subspecies and the two wild rice species was ground in liquid nitrogen to fine powder using mortar and pestle. Genomic DNAs were isolated using the Plant Genomic DNA Purification Kit (Tiangen Inc., China) and total RNAs were isolated using the RNeasy Plant Mini Kit (Qiagen Inc., Germany). DNA was fragmented by sonication with the Diagenome sonicator to a mean size of approximately 250 bp, followed by blunting, 3’-end addition of dA, and adaptor ligation according to the manufacturer’s instruction (Illumina). The bisulfite conversion of rice DNA was carried out using a modified (NH_4_)HSO_3_-based protocol [33]. Bisulfite-treated DNAs were PCR amplified with 16 cycles. The resultant DNAs were applied to paired-end sequencing with the read length of 44 or 75 nt for each end using the ultrahigh-throughput Illumina Genetic Analyzer (GA 2) as per the manufacturer’s instructions.

### Mapping and processing of BS-Seq reads

Because *japonica* rice has high quality reference genome sequence and gene annotation information, all reads from four rice samples were mapped to the Nipponbare IRGSP genome sequence (build 4 assembly), which was downloaded from RAP-DB (http://rapdblegacy.dna.affrc.go.jp/archive/build4/OsGenome_RAP2.tar.gz). Since DNA methylation has strand specificity, the plus strand and the minus strand of Nipponbare genome should be separated and used as different alignment target sequences for BS-Seq reads. That is, each cytosine in reference genome sequences was converted to thymine, termed T-genome which represents the plus strand. Meanwhile, each guanine in reference genome sequences was converted to adenosine, termed A-genome which represents the minus strand. To map the raw 44 or 75 nt pair-ended BS-Seq reads, the original reads were computationally converted to the alignment forms with the following steps: 1) observed cytosines on the forward read of each read pair were *in silico* replaced by thymines; 2) observed guanines on the reverse read of each read pair were *in silico* replaced by adenosines. The converted reads were then mapped to both strands of the A- and T- genome sequence using SOAP2 allowing up to two mismatches for 44 nt reads and four mismatches for 75 nt reads [34]. Reads mapped to the same start position for both ends were regarded as clonal duplicates, which might have been generated during PCR process, and only one of them was kept. Only reads uniquely mapped to either of the strands were then retained for further analysis. After above filtration, for methylcytosine (mC) detection, we transformed each aligned read and the two strands of the Nipponbare genome back to their original forms to build an alignment between the original forms. Cytosines in the BS-Seq reads matching the corresponding cytosines in the plus strand of the reference genome, or guanines in the BS-Seq reads matching the corresponding guanines in the minus strand of the reference genome will be regarded as potential mCs. To exclude the false positive caused by base calling process, we removed those potential mCs with Q scores lower than 20, which means that a base is correctly called at more than 99% probability, a highly conservative criterion for calling reliable bases.

We used the unmethylated chloroplast genome [21] to calculate the sum of non-conversion rate and T-C sequencing error rate, and then conducted binomial tests using these values with false positive rate below 5% to exclude those mCs that may be the results of non-conversion of cytosines during our bisulfite treatment or T to C sequencing errors during base calling process.

The method of mapping BS-seq reads has been successfully applied in profiling the silk gland methylome of silkworm and the peripheral blood mononuclear cell methylome of humans [15,35]. In these published studies as well as in this study, experimental validation confirmed the reliability of mC calling. To further compare the performance between our pipeline and other reported softwares, we chose data from one lane of *japonica* sample (17,406,765 paired reads) and the plus strand of chromosome01 to call mCs using both our method and Bismark [36] with the same parameters (because BS seeker [37] does not support paired-end reads, we could not use BS seeker to do the comparison). The results showed that in general the two methods gave very similar results. For example, 92%, 91% and 95% mCs for CG, CHG and CHH sequence context called by Bismark were also identified by our method. And the methylation levels obtained using two methods show high correlation for each sequence context (see Table 4 for details). Furthermore, our method shows slightly higher sensitivity than Bismark, which could be caused by employing different short reads aligners (our method used SOAP, while Bismark used Bowtie). Therefore, we used mCs called with our pipeline to conduct all following analyses.

**Table 4:** Comparison of mC calling between our pipeline and Bismark

	**Called by both**	**Called only by Bismark**	**Called only by our method**
**CG**	414,168	31,180	43,897
**CHG**	217,820	20,356	31,183
**CHH**	282,825	16,525	33,705

### SNPs calling using BS-Seq reads

Because DNA methylation has strand specificity, BS-Seq reads mapped to T-genome contain the DNA sequence information of plus strand, while reads mapped to A-genome contain the information of minus strand. Thus we could use BS-Seq reads to call SNPs directly for both plus and minus strands for each sample. After mapping and processing of BS-Seq reads described in above step, SNPs in both strands were called using Samtools [38]. To get high-quality SNPs, we used strict standards for our results—only bases with base quality (Q score) of at least 30 and positions covered by at least 5 reads were used for our SNP calling process. Although bisulfite treatment will convert C to T in both strands which will affect judgment of some kinds of genotypes during SNPs calling process, we can use SNP information from both strands to solve this problem. Some genotypes (A/G, G/A, C/A, C/G, T/A, T/G, A/A, G/G, the former indicates reference base and latter indicates sample’s base) can be correctly called using results from plus strand, while the other genotypes (A/C, A/T, G/T, G/C, C/T, T/C, T/T, C/C) can be correctly called using results from minus strand. Under this principle, a total of 37,141, 50,340, 65,680, and 83,594 high-quality SNPs in *japonicaindicaO. rufipogon*, and *O. nivara* were obtained respectively and used for further analysis.

### Validation of BS-Seq results

To verify the BS-Seq results, we picked 6 regions, including 4 promoter regions (Os12g0264800, Os01g0116800, Os01g0543000, and Os04g0431700 genes) and 2 TTR regions (Os11g0111101 and Os08g0150200 genes), to validate the methylation status in all the four samples using the traditional bisulfite sequencing method for single genes.

### Digital Gene Expression (DGE) tag libraries construction

DGE-tag libraries were constructed from the total RNAs isolated from the same four rice panicles used for extracting DNA with the DGE-Tag Profiling NlaIII Sample Prep Kit (Illumina) according to the manufacturer’s instructions.

### Mapping and processing of Digital Gene Expression (DGE) tags

Full-length cDNA sequences of rice genes were downloaded from RAP-DB (http://rapdblegacy.dna.affrc.go.jp/archive/build4/rep_RAP2.tar.gz). A total of 24,955 genes supported with full length cDNA were used for DGE analysis. All possible CATG + 17nt tag sequences were created from 24,955 full-length cDNAs and used as the reference tag database. Unique tag sequences and their numbers were extracted from our raw DGE tags and these tags were aligned against the reference tag database using SOAP [39]. Only perfect matches were kept for further analysis. Within genes, most of the DGE tags were mapped to the most 3’-end CATG sites of genes (Additional file 15), suggesting that transcriptional termination sites of most rice genes we used were annotated correctly and that performing DGE analysis for those genes was suitable and reliable. The expression level of a gene was represented by the total number of tags that uniquely aligned to that gene. Gene expression levels were normalized to tag number per million tags for gene expression comparisons among different samples.

### Methylation level, TE and smRNA density analyses

Annotation of TEs was downloaded from RAP-DB (http://rapdblegacy.dna.affrc.go.jp/archive/build4/OsNIAS_b4_chromOut.tar.gz) and smRNA sequences were downloaded from rice MPSS database (http://mpss.udel.edu/data-files/rice/small/smallRNA_summary.txt). Sequences of smRNAs were mapped to the rice reference genome using SOAP [39] without allowing mismatch, and uniquely mapped smRNAs were used for further analysis. Methylation level refers to the proportion of reads showing mC among all reads covering the same cytosine site. It can further be classified as absolute methylation level (total methylation level of mCs divided by the total sequence length of the calculated region) and relative methylation level (total methylation level of mCs divided by total number of cytosine sites in the calculated region), both of which were used for our analysis. TE or smRNA density was defined as the ratio of the number of bases belonging to TEs or smRNAs to the total length of the calculated region.

### Methylation-expression correlation analysis for *Arabidopsis*

The single-base methylation profile and corresponding gene expression profile of *Arabidopsis* were downloaded from NCBI SRA (Sequence Read Archive) database (http://www.ncbi.nlm.nih.gov/sra, the accession numbers are SRA000284 for BS-Seq, also referred as MethylC-Seq in Lister *et. al.*’s paper [13], and SRA000286 for mRNA-seq). The genome sequences and gene annotation information (TAIR9) were downloaded from the ftp site of TAIR (The *Arabidopsis* Information Resource, ftp://ftp.arabidopsis.org/home/tair/). The method of mapping and processing of BS-Seq reads is the same as that used in rice. Mapping and processing of mRNA-seq reads were conducted using TopHat software [40]. Gene expression levels, measured by reads per kilobase of transcript per million reads (RPKM), was also calculated using TopHat. Methylation-expression correlation analyses for *Arabidopsis* were performed with the same methods as used for rice.

### Construction of methylation, genomic and expression trees

For methylation tree construction, we first calculated the absolute methylation level in sliding windows of 50 kb with steps of 25 kb across the whole genome for the four samples, and then clustered the samples based on pairwise Spearman correlation coefficients estimated from the above whole genome sliding methylation level matrix. Methylation levels of all mCs with ≥ 5 × coverage of the whole genome were also used to calculate the pairwise Spearman correlation coefficients among samples, which in turn produced similar methylation tree. High-quality SNPs called from BS-Seq reads were used to construct genomic tree among the four samples using *p* distance and neighbor-joining method implemented in MEGA [41,42]. Finally, an expression tree was constructed using pairwise Spearman expression correlation coefficients among the four samples based on DGE data. Methylation and expression trees were constructed using the hclust function of R statistical software (http://www.r-project.org/).

### Examination of the relationship between genetic divergence and methylation divergence

To examine the relationship between genetic and methylation divergence, the average number of nucleotide differences per site among samples for different genomic regions was calculated using SNPs obtained from the above step with 50 kb sliding window and a step of 25 kb through the whole genome, which was used to measure genetic divergence. Then average Spearman correlation coefficients of methylation level of all cytosines with ≥ 5 × coverage among samples were calculated for same sliding windows and used to measure methylation divergence in different genomic regions.

### Identification of differentially methylated genes between cultivated and wild rice

Because mCs in promoters, TTRs and gene bodies have significant effects on gene expression, we identified genes with different methylation status in these three regions among the four rice samples. Only regions with above 80% sequencing coverage were used for further analysis. Methylation levels of all Cs within promoters/TTRs/gene-bodies were calculated and used to perform two-sample Wilcoxon rank sum tests between any samples. Differentially-methylated genes were identified for each pairwise comparison using a significance level of *alpha* < 0.05. Genes that are significantly methylation-upregulated or methylation-downregulated consistently in all cultivated vs. wild rice pairwise comparisons but are not significantly different within cultivated or wild rice comparisons were respectively identified as methylation-upregulated or methylation-downregulated genes between cultivated and wild rice.

### Accession codes

The methylome data have been deposited into the NCBI Short Read Archive (SRA, http://www.ncbi.nlm.nih.gov/sra/) under accession number SRA012190 and DGE data have been deposited into the NCBI Gene Expression Omnibus (GEO, http://www.ncbi.nlm.nih.gov/geo) under accession number GSE20871.

## Abbreviations

TTRs, transcriptional termination regions; BS-Seq, bisulfite-treated genomic DNA sequencing; mC, methylated cytosine; TEs, transposable elements; DGE, Digital Gene Expression tag profiling; SAGE, Serial Analysis of Gene Expression; FL-cDNAs, nonredundant full-length cDNAs; CV, coefficient of variation.

## Competing interests

The authors declare that they have no competing interests.

## Authors’ contributions

WW, JW, JZ and SG designed the study. XL, WW, HX, XX, XC and JW wrote the manuscript. FH, XZ, SG, SZ and QL collected the samples. JZ, JY, XZ and HX constructed the BS-Seq libraries and conducted the BS validation. MY, GZ, RL, CY performed the Illumina sequencing. XL, HX, FH, and XX performed the data analyses. All authors read and approved the final manuscript.

## Supplementary Material

Additional file 1 **Distribution of mCs on the sense and antisense strands of rice chromosomes for each sequence context in other samples (a) indica. (b) O. rufipogon. (c) O. nivara.** The sliding window size is 50 kb and the step size is 25 kb. The black circle indicates the centromeric position of a chromosome. Some centromeric regions of chromosomes have not been completely sequenced and thus are displayed as gaps in the figures. Click here for file

Additional file 2 Relationships between methylation level and sequence length in genes (left) and TE regions (right) in indica (a), O. rufipogon (b), and O. nivara (c), in which both absolute (top) and relative (bottom) methylation levels were analyzed. Click here for file

Additional file 3 **Relationships between gene expression and methylation in different genic regions for indica (a-b), O. rufipogon (c-d), and O. nivara (e-f).** For panel a, c and e, genes are categorized into unmethylated (black line) and methylated ones, and the latter were further divided into five groups based on methylation level (from Group 1 of the 20% of genes with the lowest methylation to Group 5 of the x20% with the highest methylation level). For panel b, d and f, methylation-expression Spearman correlation coefficients along genes and their 2 kb-flanking regions were displayed. The correlation coefficients were calculated using an overlapping sliding window of 5% of sequence length at a step of 2.5% of sequence length. Click here for file

Additional file 4 **Relationships between gene expression and methylation in different genic regions and sequence contexts.**(a) Promoter methylation. (b) TTR methylation. (c) Gene body methylation. Methylation was measured using absolute methylation level (total methylation level of mCs divided by sequence length of the calculated region). Genes are categorized into unmethylated (black line) and methylated ones, and the latter were further divided into five groups based on methylation level (from Group 1 of the 20% of genes with the lowest methylation to Group 5 of the 20% with the highest methylation level). Gene expression level was measured by log2 value of its tag number and is indicated on the x axis. The fraction for each group of methylated and unmethylated genes is shown on the y-axis. Click here for file

Additional file 5 **Methylation level distributions in gene body and 2-kb flanking sequences in Arabidopsis.** Absolute (a) and relative (b) methylation level distributions in gene body and 2-kb flanking sequences in Arabidopsis. Methylation levels along gene body and their 2 kb-flanking regions were calculated using an overlapping sliding window of 5% of sequence length at a step of 2.5% of sequence length. The related raw data for Arabidopsis were downloaded from http://www.ncbi.nlm.nih.gov/sra(SRA000284 Click here for file

Additional file 6 Coefficient of variation (CV) of methylation level for methylcytosine among species in different functional elements. Click here for file

Additional file 7 **Coefficient of variation (CV) of methylation level for methylcytosine among species in different functional elements and sequence context in different methylation level groups.** (a) CG context (b) CHG context (c) CHH context. Click here for file

Additional file 8 List of differentially methylated genes between cultivated and wild rice. Click here for file

Additional file 9 **Validation results for the promoter region of Os12g0264800 gene using the traditional bisulfite sequencing method.**In each panel, the top histogram shows the validation results from traditional bisulfite sequencing and the bottom shows the BS-Seq results. Methylation level of individual cytosine sites located on the genome (indicated on the x-axis) is shown on the y-axis. (a) CG context. (b) CHG context. (c) CHH context. Click here for file

Additional file 10 **Validation results of TTR region of Os11g0111101 gene by traditional bisulfite sequencing.** In each panel, the top histogram shows the validation results from traditional bisulfite sequencing and the bottom shows the BS-Seq results. Methylation level of individual cytosine sites located on the genome (indicated on the x-axis) is shown on the y-axis. (a) CG context. (b) CHG context. (c) CHH context. Click here for file

Additional file 11 **Validation results of promoter region of Os01g0116800 gene by traditional bisulfite sequencing. **In each panel, the top histogram shows the validation results from traditional bisulfite sequencing and the bottom shows the BS-Seq results. Methylation level of individual cytosine sites located on the genome (indicated on the x-axis) is shown on the y-axis. (a) CG context. (b) CHG context. (c) CHH context. Click here for file

Additional file 12 **Validation results of promoter region of Os01g0543000 gene by traditional bisulfite sequencing.** In each panel, the top histogram shows the validation results from traditional bisulfite sequencing and the bottom shows the BS-Seq results. Methylation level of individual cytosine sites located on the genome (indicated on the x-axis) is shown on the y-axis. (a) CG context. (b) CHG context. (c) CHH context. Click here for file

Additional file 13 **Validation results of promoter region of Os04g0431700 gene by traditional bisulfite sequencing.** In each panel, the top histogram shows the validation results from traditional bisulfite sequencing and the bottom shows the BS-Seq results. Methylation level of individual cytosine sites located on the genome (indicated on the x-axis) is shown on the y-axis. (a) CG context. (b) CHG context. (c) CHH context. Click here for file

Additional file 14 **Validation results of TTR region of Os08g0150200 gene by traditional bisulfite sequencing.** In each panel, the top histogram shows the validation results from traditional bisulfite sequencing and the bottom shows the BS-Seq results. Methylation level of individual cytosine sites located on the genome (indicated on the x-axis) is shown on the y-axis. (a) CG context. (b) CHG context. (c) CHH context. Click here for file

Additional file 15 **Distribution of DGE tags across CATG sites within genes and gene numbers supported by DGE tags from different CATG sites.** (a) japonica. (b) indica. (c) O. rufipogon. (d) O. nivara. For each panel, x-axis indicates CATG site positions from 3’-end of genes, while y-axis in left indicates the corresponding numbers of total tags mapped to different CATG positions and y-axis in right indicates the corresponding gene numbers supported by DGE tags mapped to different CATG sites. Click here for file
