# Supplementary figures and images for: Single-base resolution maps of cultivated and wild rice methylomes and regulatory roles of DNA methylation in plant gene expression
Source: BMC Genomics. 2012 Jul 2;13:300. doi: 10.1186/1471-2164-13-300 (PMC3447678; doi:10.1186/1471-2164-13-300)

(a)

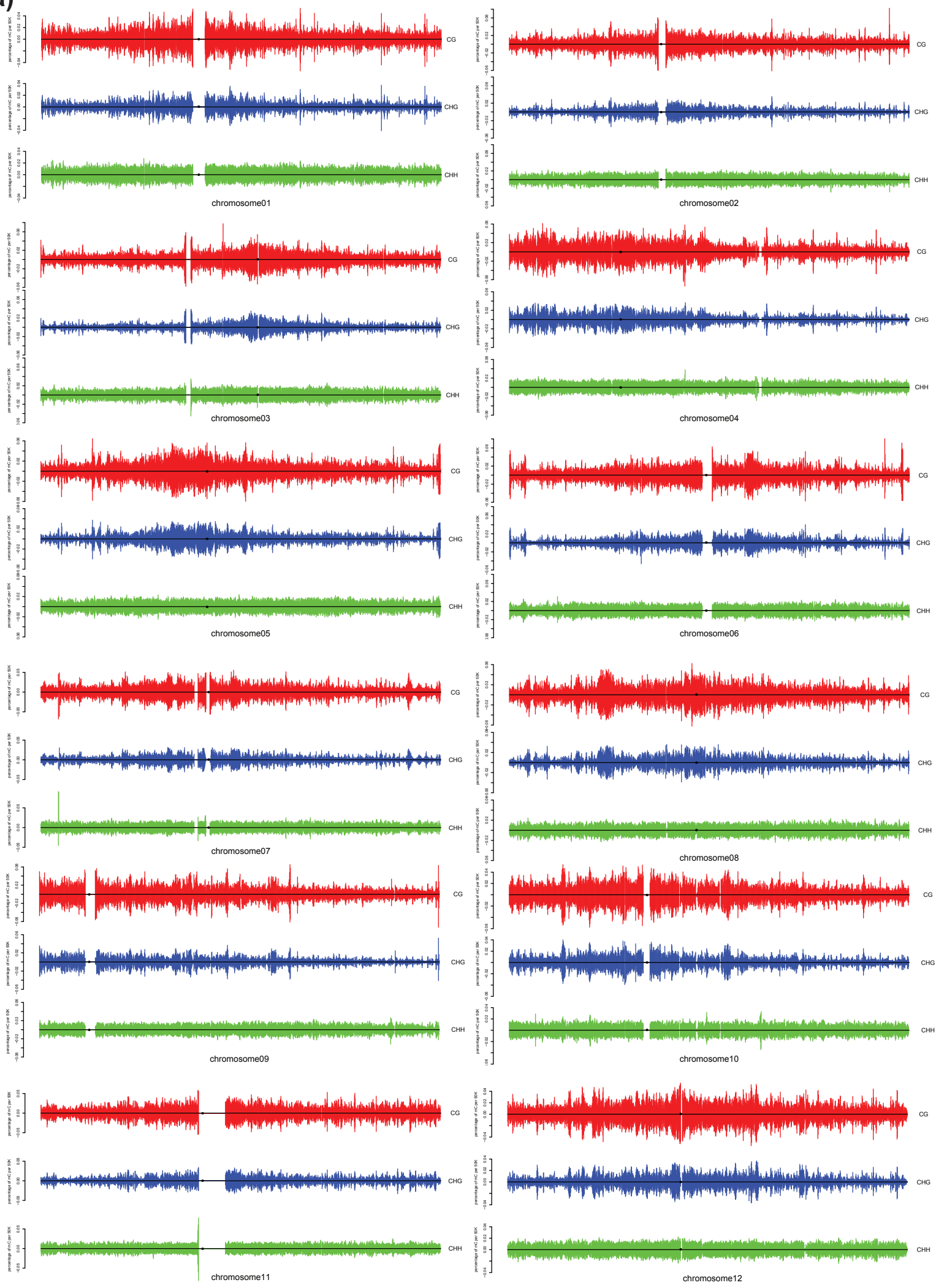

**(b)**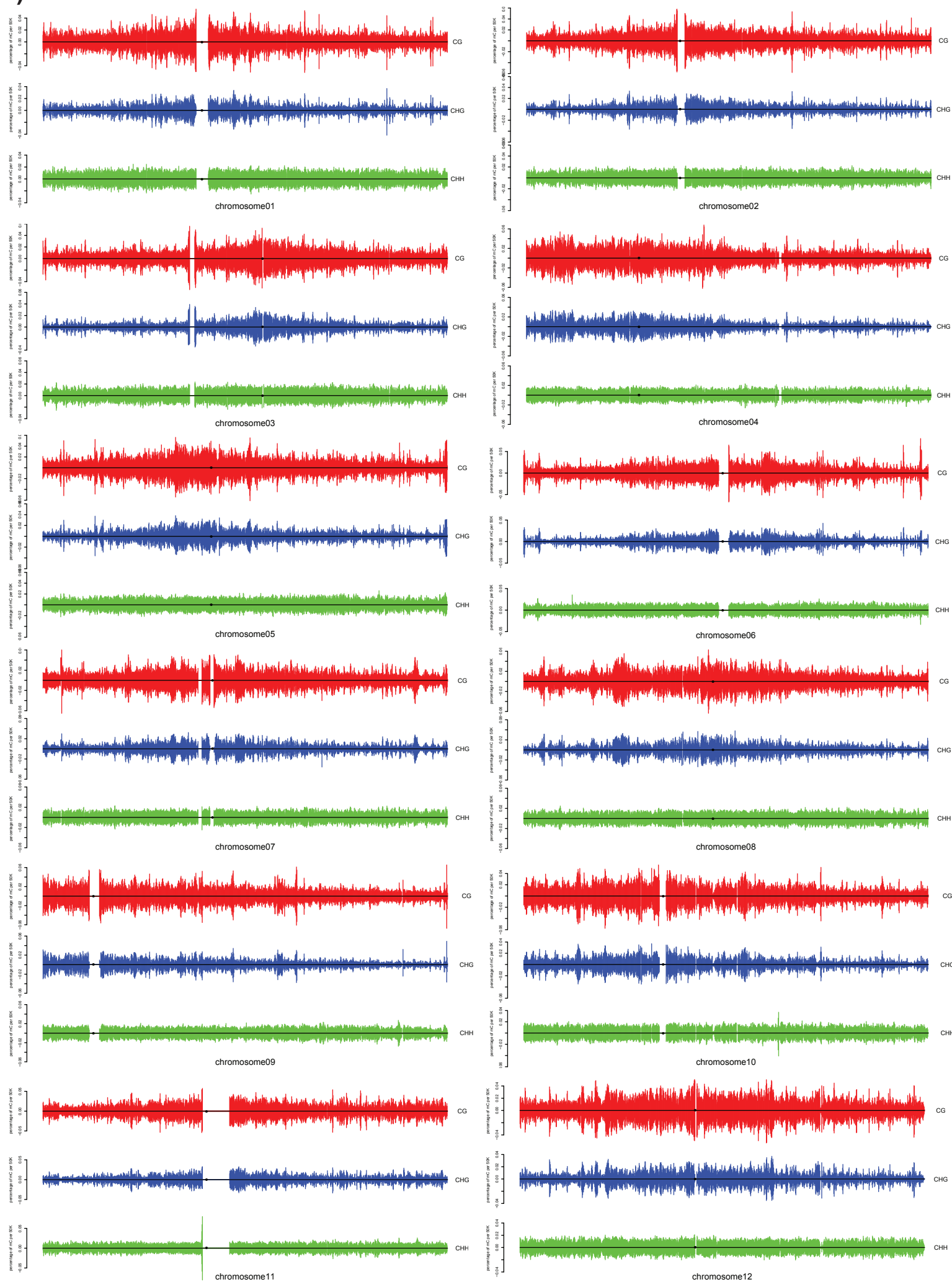

(c)

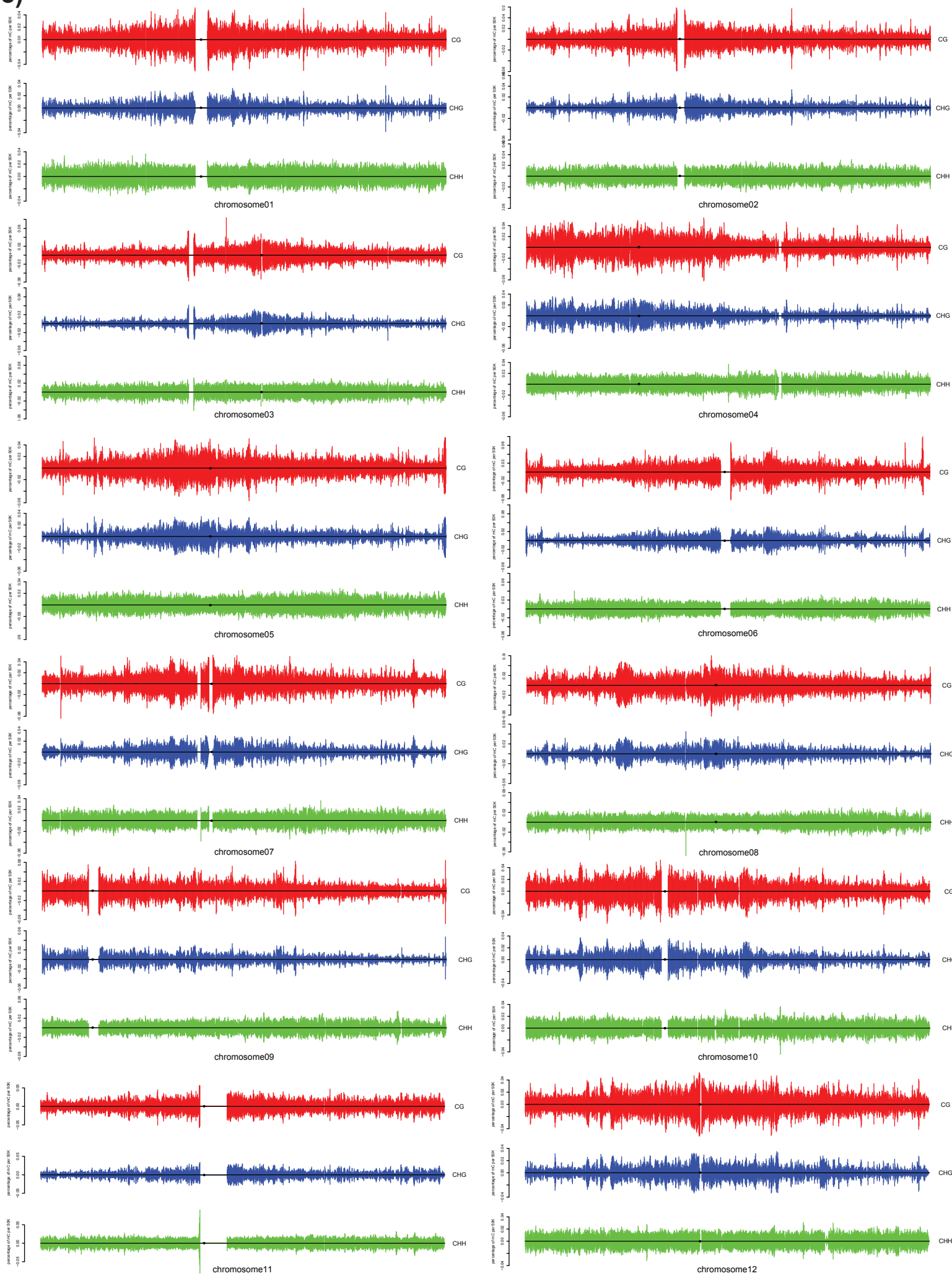

Supplement: Additional file 1 — Distribution of mCs on the sense and antisense strands of rice chromosomes for each sequence context in other samples (a) indica. (b) O. rufipogon. (c) O. nivara. The sliding window size is 50 kb and the step size is 25 kb. The black circle indicates the centromeric position of a chromosome. Some centromeric regions of chromosomes have not been completely sequenced and thus are displayed as gaps in the figures. [file 1471-2164-13-300-S1.pdf]

(a)

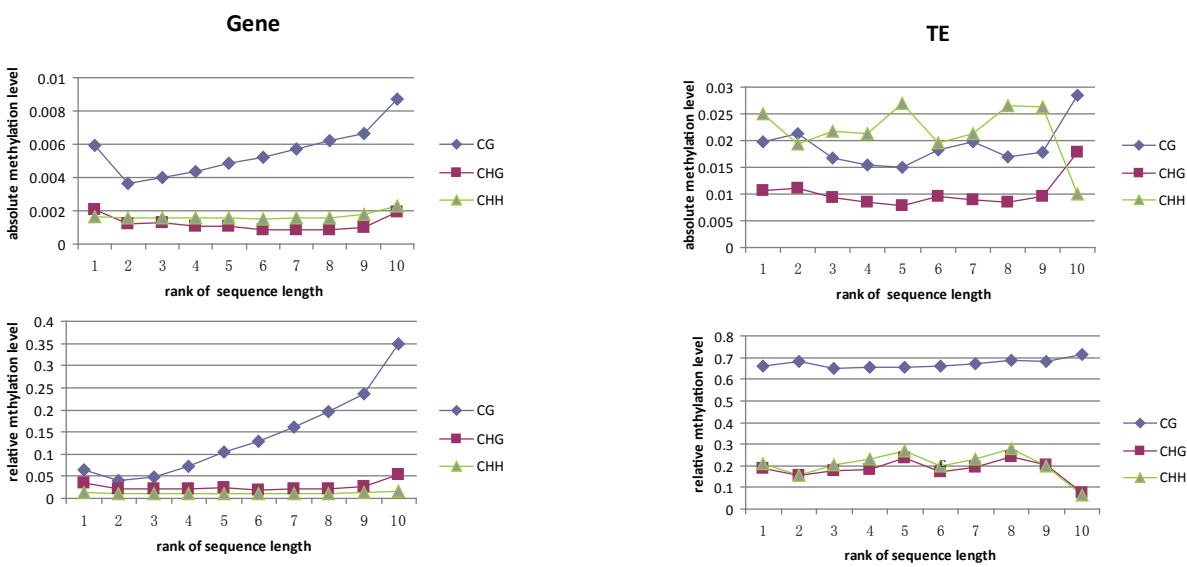

(b)

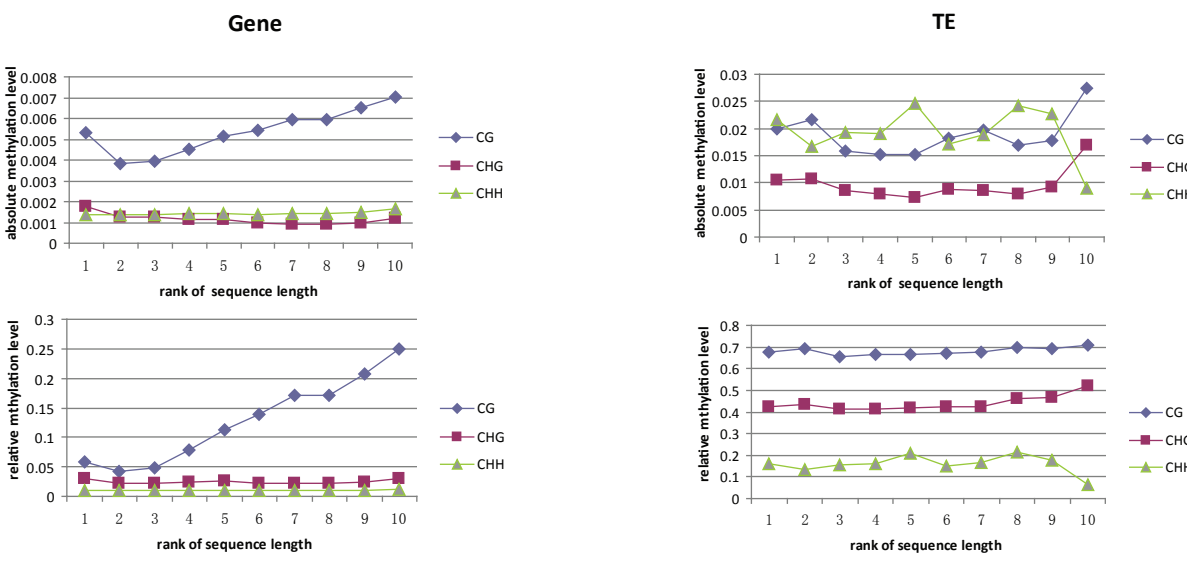

(c)

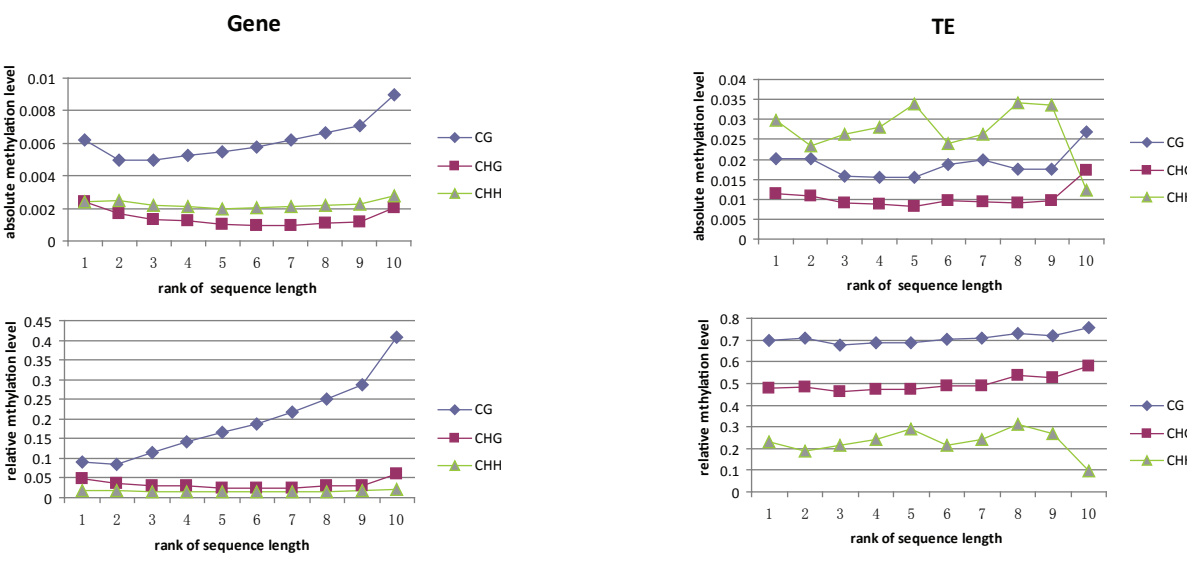

Supplement: Additional file 2 — Relationships between methylation level and sequence length in genes (left) and TE regions (right) in indica (a), O. rufipogon (b), and O. nivara (c), in which both absolute (top) and relative (bottom) methylation levels were analyzed. [file 1471-2164-13-300-S2.pdf]

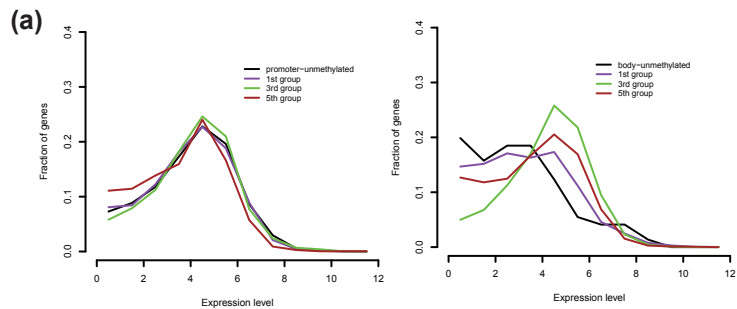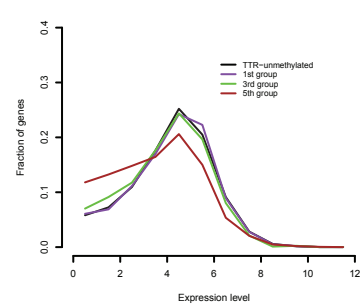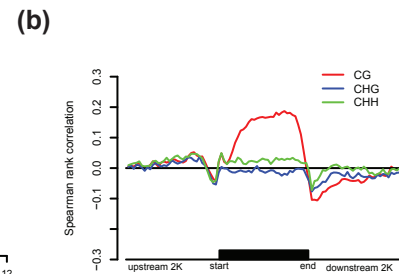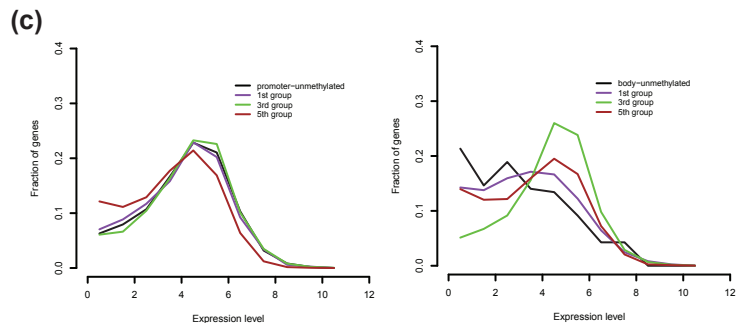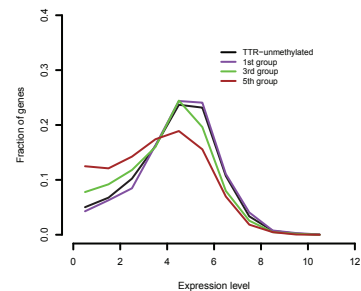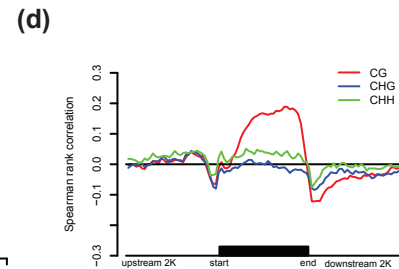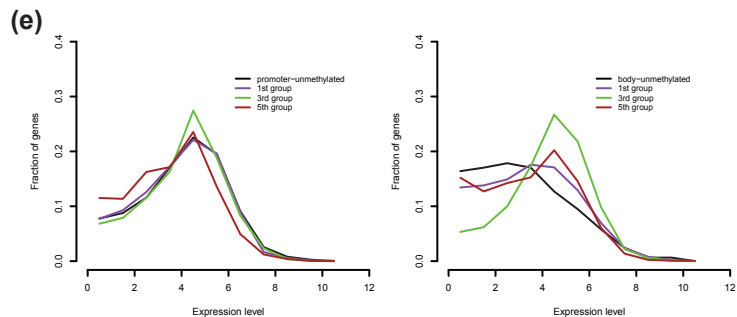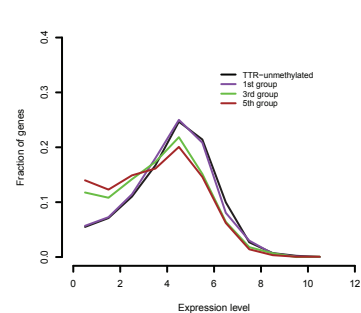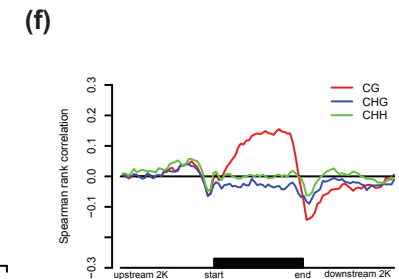

Supplement: Additional file 3 — Relationships between gene expression and methylation in different genic regions for indica (a-b), O. rufipogon (c-d), and O. nivara (e-f). For panel a, c and e, genes are categorized into unmethylated (black line) and methylated ones, and the latter were further divided into five groups based on methylation level (from Group 1 of the 20% of genes with the lowest methylation to Group 5 of the x20% with the highest methylation level). For panel b, d and f, methylation-expression Spearman correlation coefficients along genes and their 2 kb-flanking regions were displayed. The correlation coefficients were calculated using an overlapping sliding window of 5% of sequence length at a step of 2.5% of sequence length. [file 1471-2164-13-300-S3.pdf]

**(a)**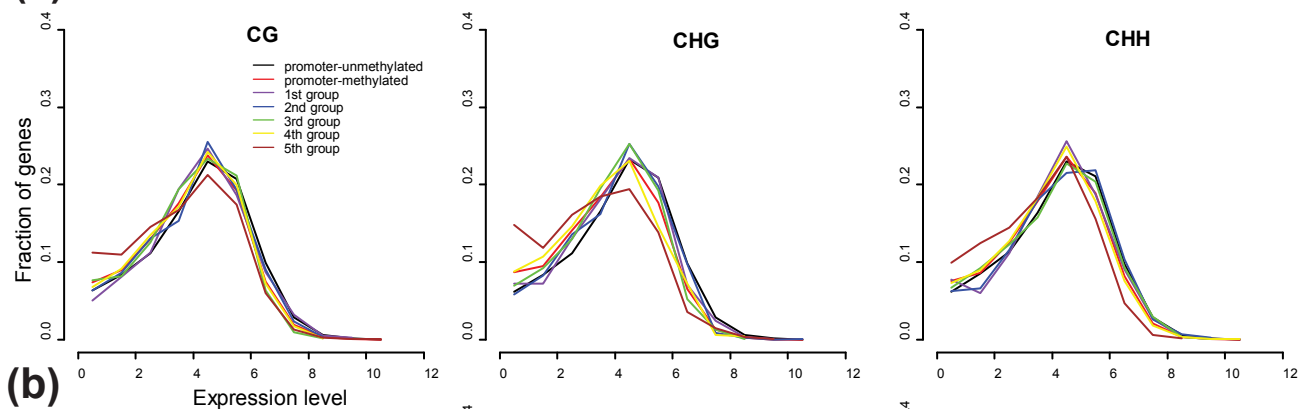**(b)**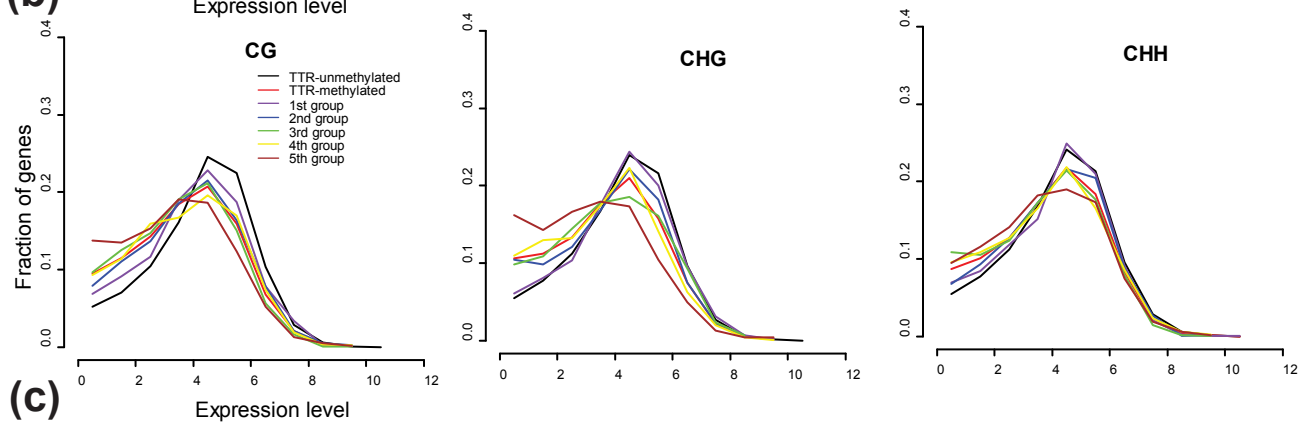**(c)**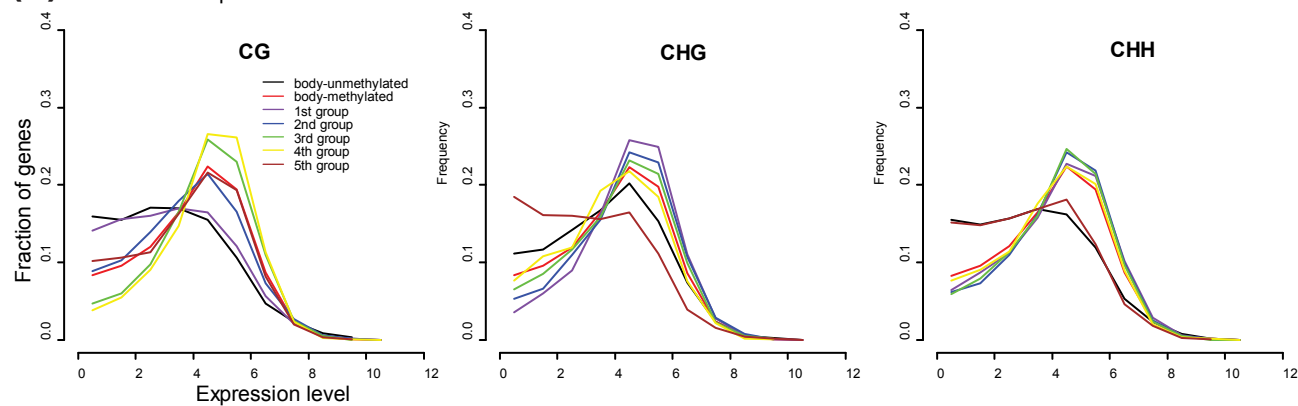

Supplement: Additional file 4 — Relationships between gene expression and methylation in different genic regions and sequence contexts.(a) Promoter methylation. (b) TTR methylation. (c) Gene body methylation. Methylation was measured using absolute methylation level (total methylation level of mCs divided by sequence length of the calculated region). Genes are categorized into unmethylated (black line) and methylated ones, and the latter were further divided into five groups based on methylation level (from Group 1 of the 20% of genes with the lowest methylation to Group 5 of the 20% with the highest methylation level). Gene expression level was measured by log2 value of its tag number and is indicated on the x axis. The fraction for each group of methylated and unmethylated genes is shown on the y-axis. [file 1471-2164-13-300-S4.pdf]

**(a)**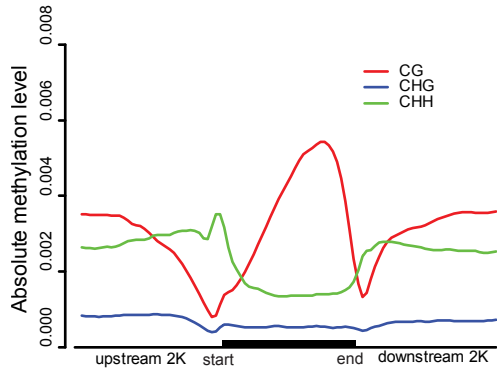**(b)**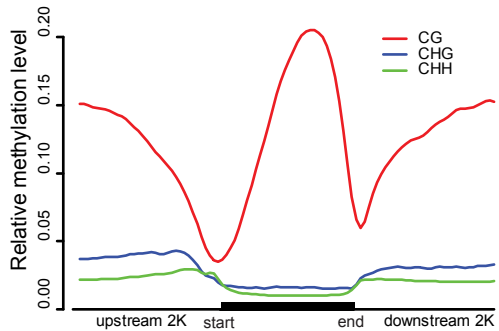

Supplement: Additional file 5 — Methylation level distributions in gene body and 2-kb flanking sequences in Arabidopsis. Absolute (a) and relative (b) methylation level distributions in gene body and 2-kb flanking sequences in Arabidopsis. Methylation levels along gene body and their 2 kb-flanking regions were calculated using an overlapping sliding window of 5% of sequence length at a step of 2.5% of sequence length. The related raw data for Arabidopsis were downloaded from http://www.ncbi.nlm.nih.gov/sra(SRA000284 [file 1471-2164-13-300-S5.pdf]

**(a)****CG**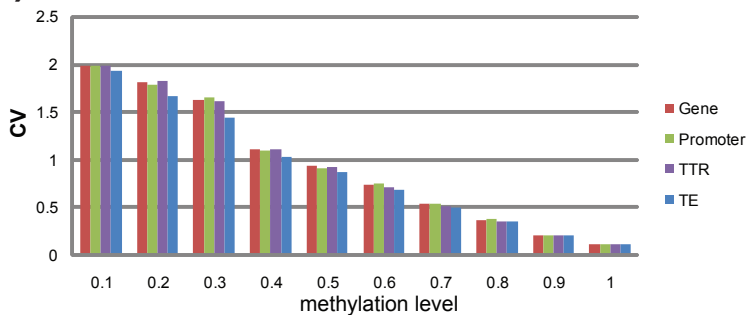**(b)****CHG**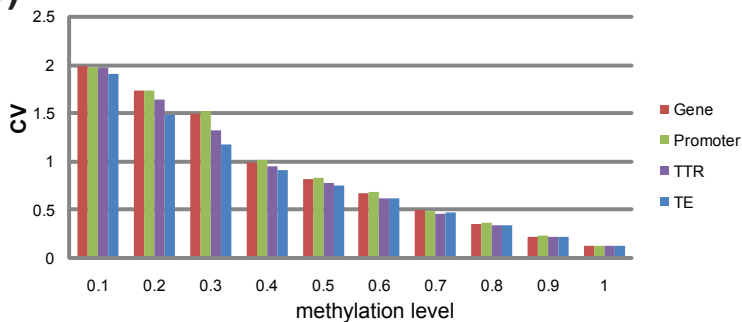**(c)****CHH**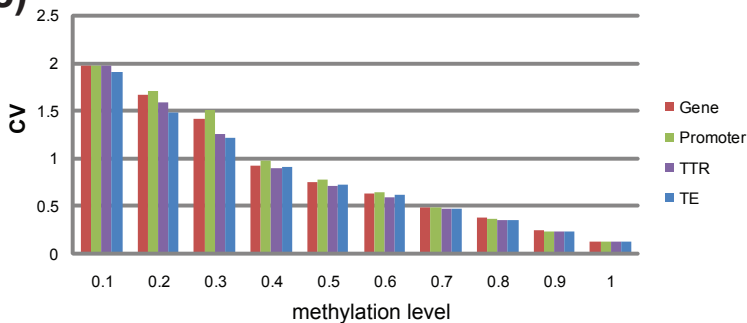

Supplement: Additional file 7 — Coefficient of variation (CV) of methylation level for methylcytosine among species in different functional elements and sequence context in different methylation level groups. (a) CG context (b) CHG context (c) CHH context. [file 1471-2164-13-300-S7.pdf]

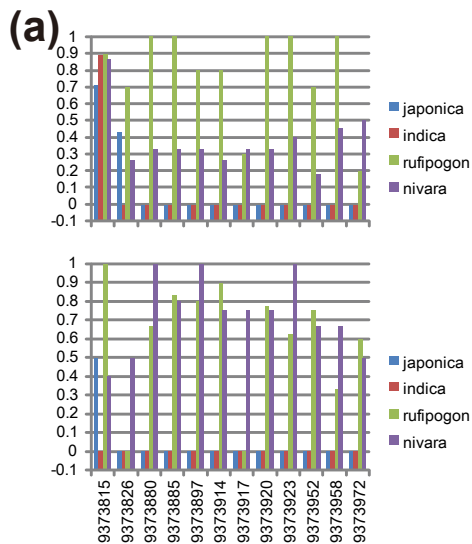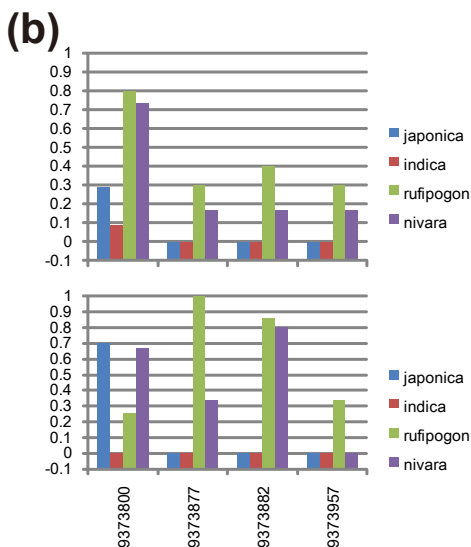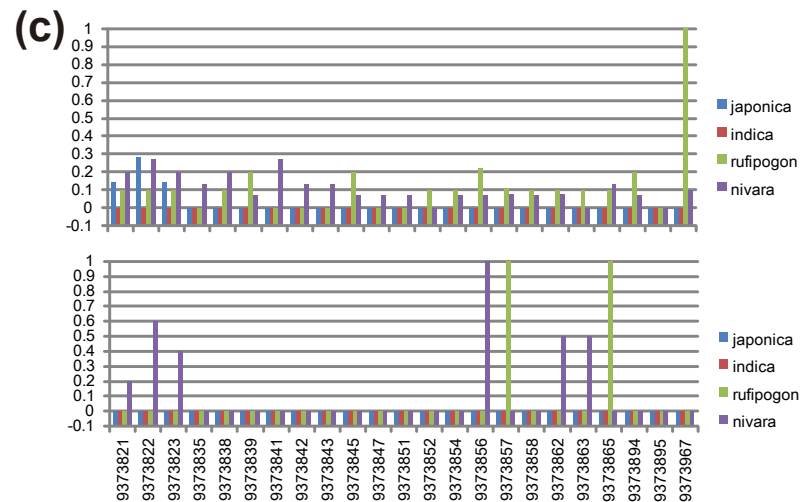

Supplement: Additional file 9 — Validation results for the promoter region of Os12g0264800 gene using the traditional bisulfite sequencing method.In each panel, the top histogram shows the validation results from traditional bisulfite sequencing and the bottom shows the BS-Seq results. Methylation level of individual cytosine sites located on the genome (indicated on the x-axis) is shown on the y-axis. (a) CG context. (b) CHG context. (c) CHH context. [file 1471-2164-13-300-S9.pdf]

**(a)**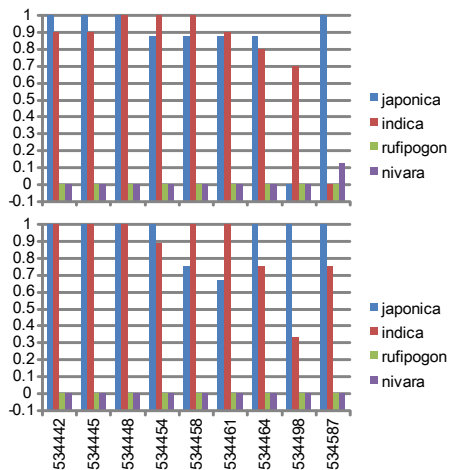**(b)**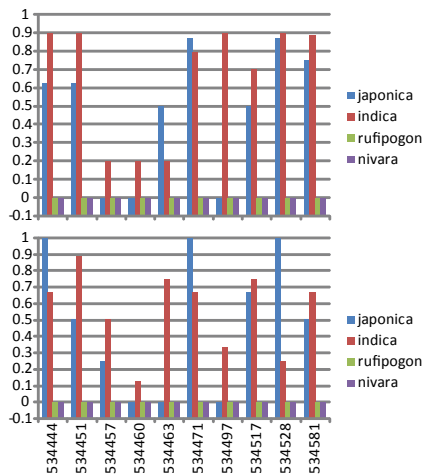**(c)**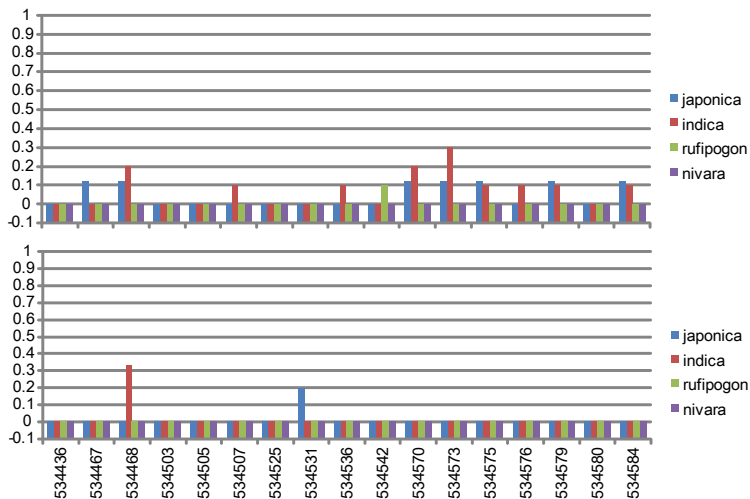

Supplement: Additional file 10 — Validation results of TTR region of Os11g0111101 gene by traditional bisulfite sequencing. In each panel, the top histogram shows the validation results from traditional bisulfite sequencing and the bottom shows the BS-Seq results. Methylation level of individual cytosine sites located on the genome (indicated on the x-axis) is shown on the y-axis. (a) CG context. (b) CHG context. (c) CHH context. [file 1471-2164-13-300-S10.pdf]

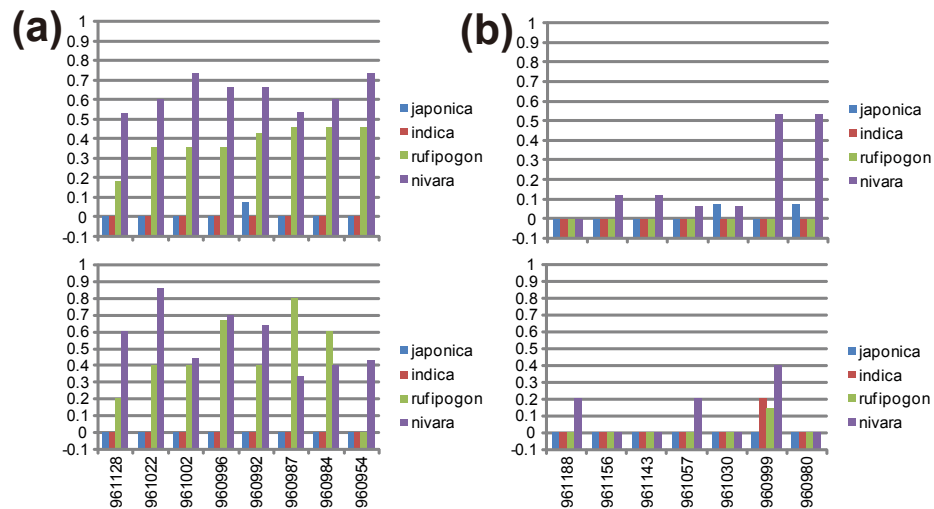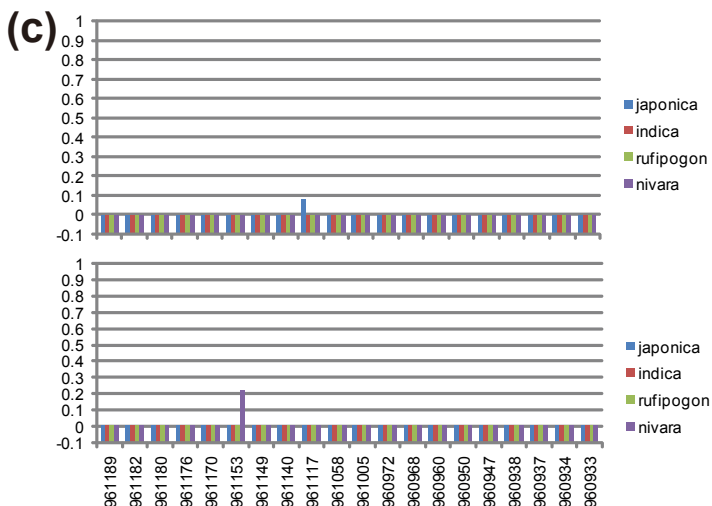

Supplement: Additional file 11 — Validation results of promoter region of Os01g0116800 gene by traditional bisulfite sequencing. In each panel, the top histogram shows the validation results from traditional bisulfite sequencing and the bottom shows the BS-Seq results. Methylation level of individual cytosine sites located on the genome (indicated on the x-axis) is shown on the y-axis. (a) CG context. (b) CHG context. (c) CHH context. [file 1471-2164-13-300-S11.pdf]

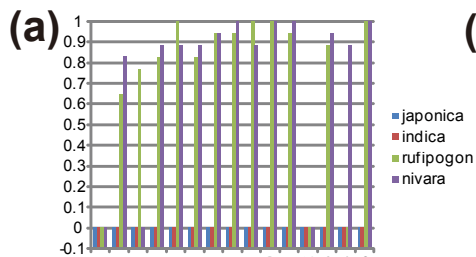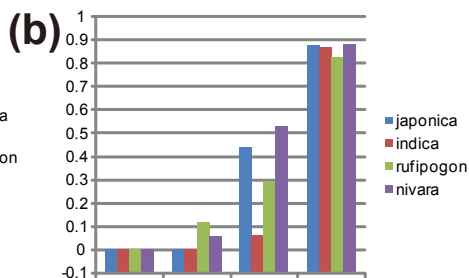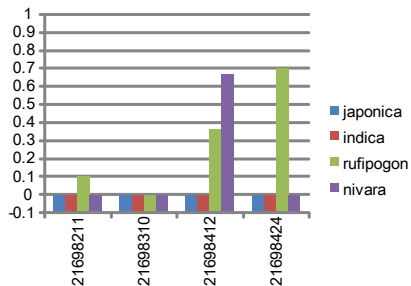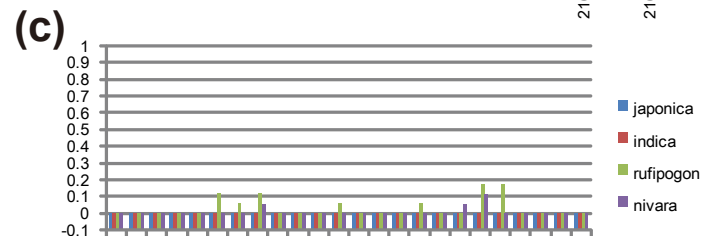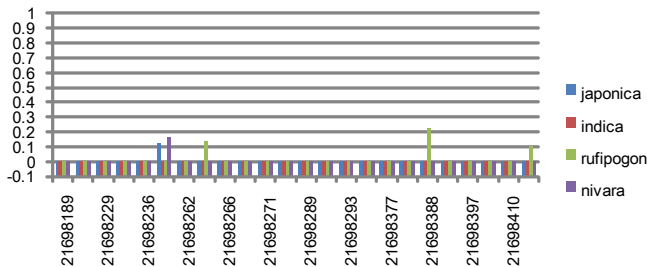

Supplement: Additional file 12 — Validation results of promoter region of Os01g0543000 gene by traditional bisulfite sequencing. In each panel, the top histogram shows the validation results from traditional bisulfite sequencing and the bottom shows the BS-Seq results. Methylation level of individual cytosine sites located on the genome (indicated on the x-axis) is shown on the y-axis. (a) CG context. (b) CHG context. (c) CHH context. [file 1471-2164-13-300-S12.pdf]

**(a)**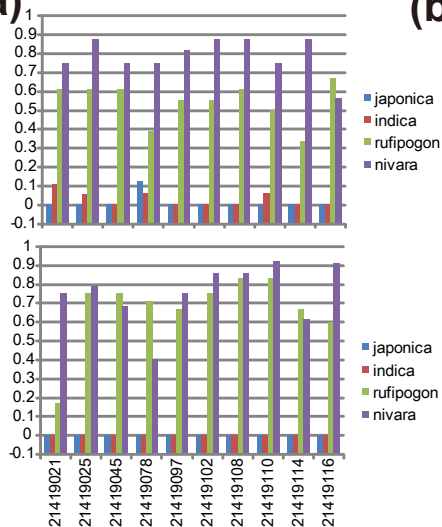**(b)**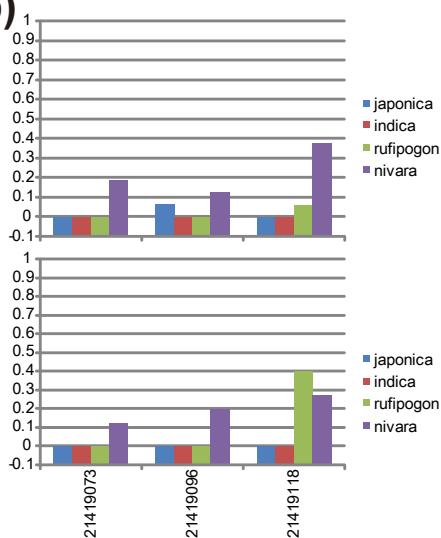**(c)**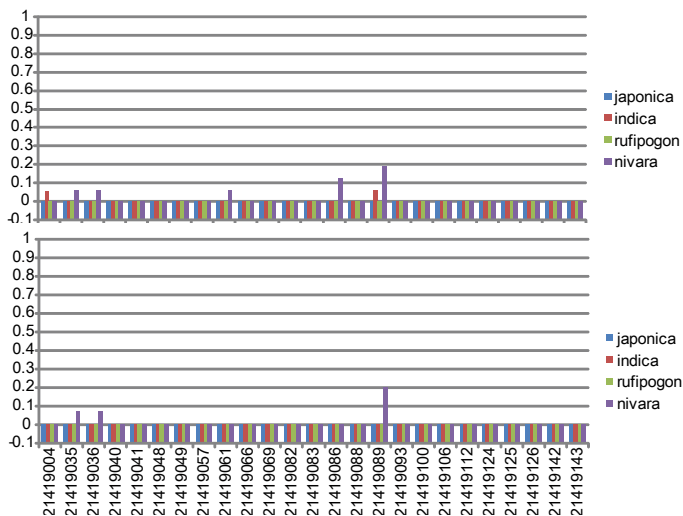

Supplement: Additional file 13 — Validation results of promoter region of Os04g0431700 gene by traditional bisulfite sequencing. In each panel, the top histogram shows the validation results from traditional bisulfite sequencing and the bottom shows the BS-Seq results. Methylation level of individual cytosine sites located on the genome (indicated on the x-axis) is shown on the y-axis. (a) CG context. (b) CHG context. (c) CHH context. [file 1471-2164-13-300-S13.pdf]

**(a)**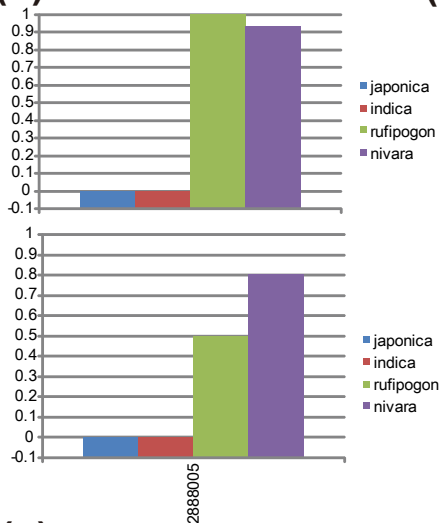**(b)**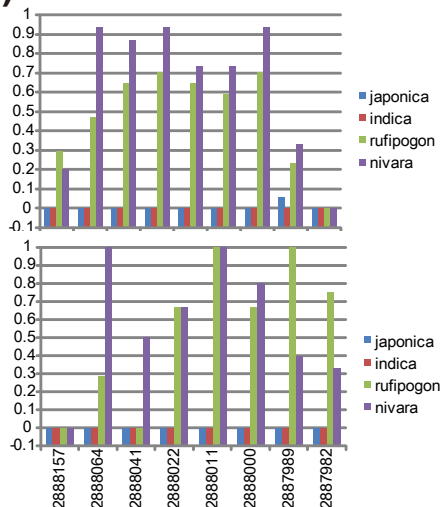**(c)**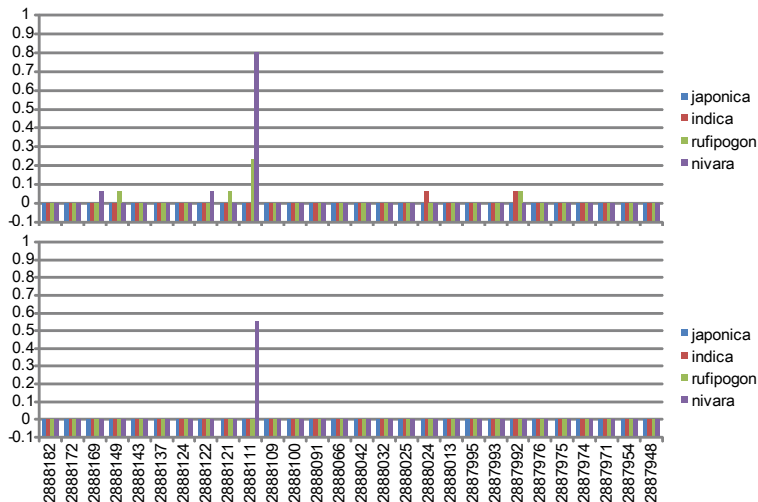

Supplement: Additional file 14 — Validation results of TTR region of Os08g0150200 gene by traditional bisulfite sequencing. In each panel, the top histogram shows the validation results from traditional bisulfite sequencing and the bottom shows the BS-Seq results. Methylation level of individual cytosine sites located on the genome (indicated on the x-axis) is shown on the y-axis. (a) CG context. (b) CHG context. (c) CHH context. [file 1471-2164-13-300-S14.pdf]

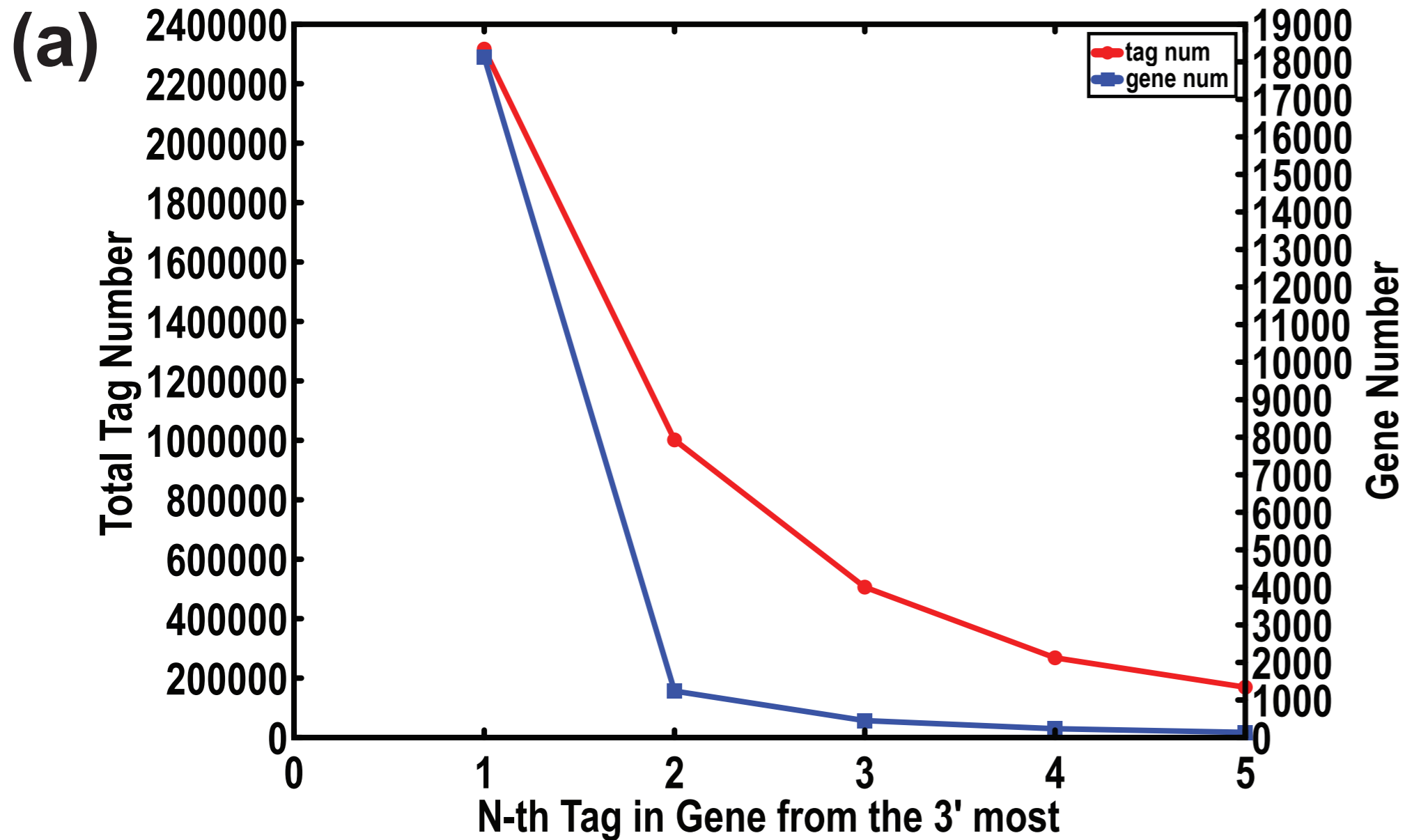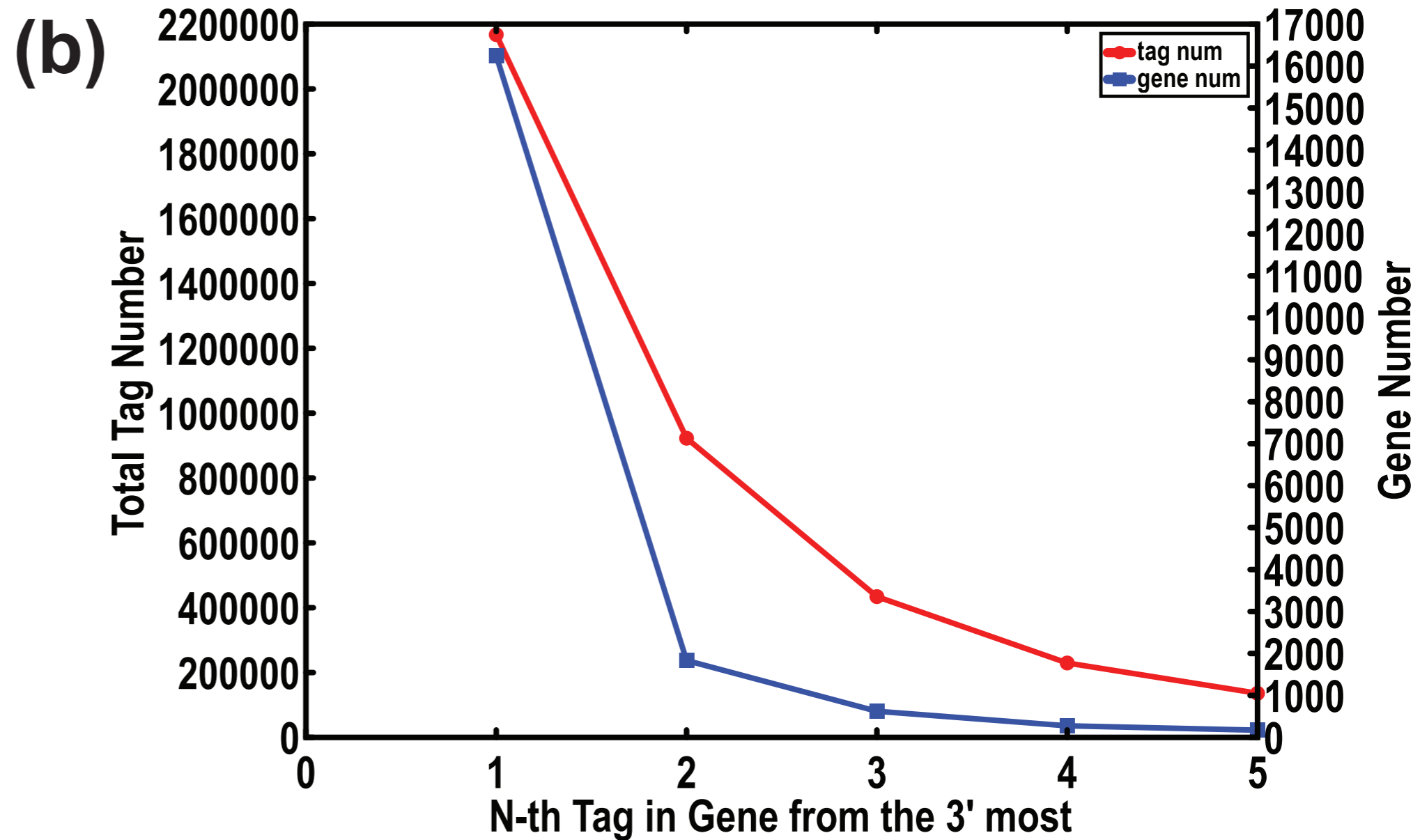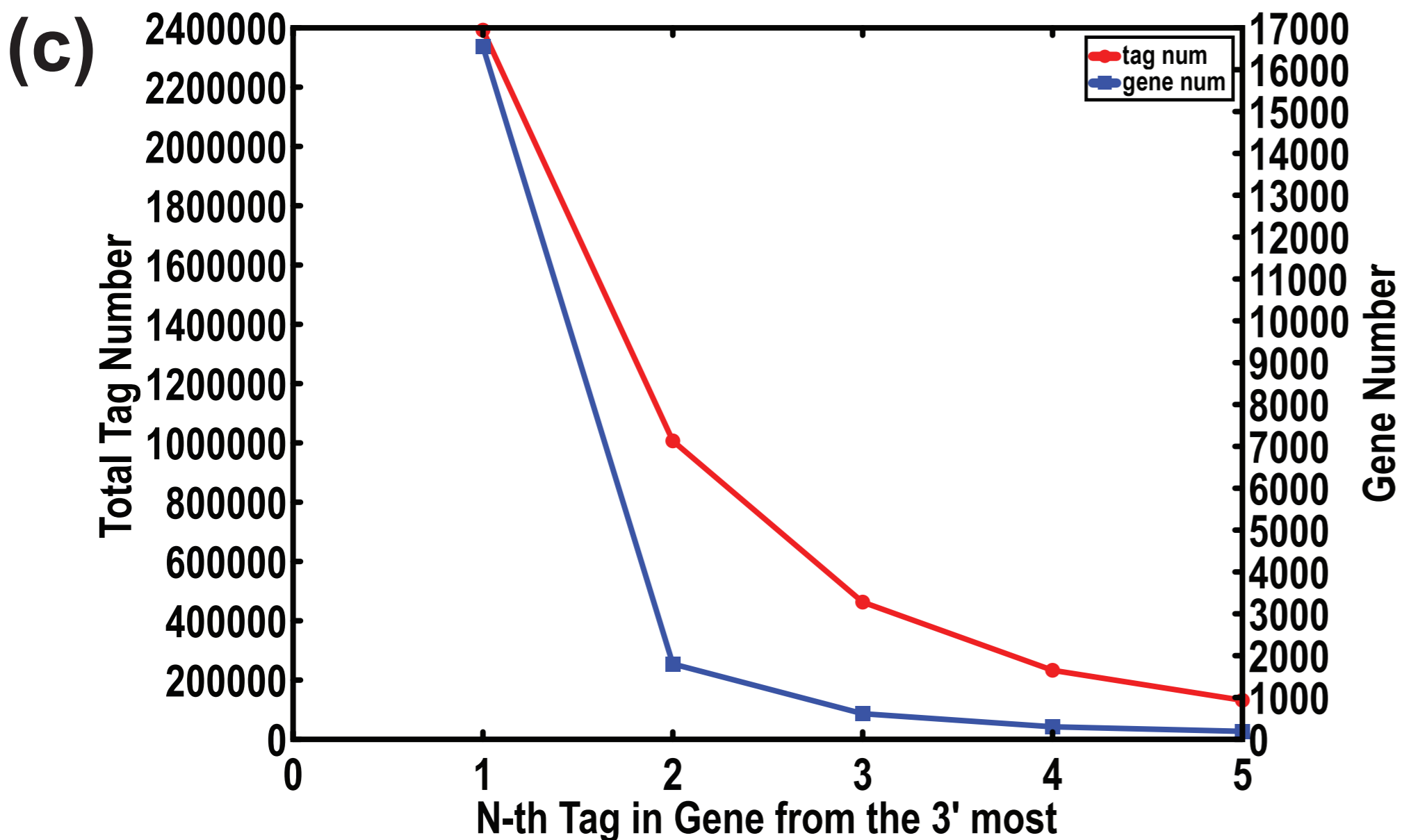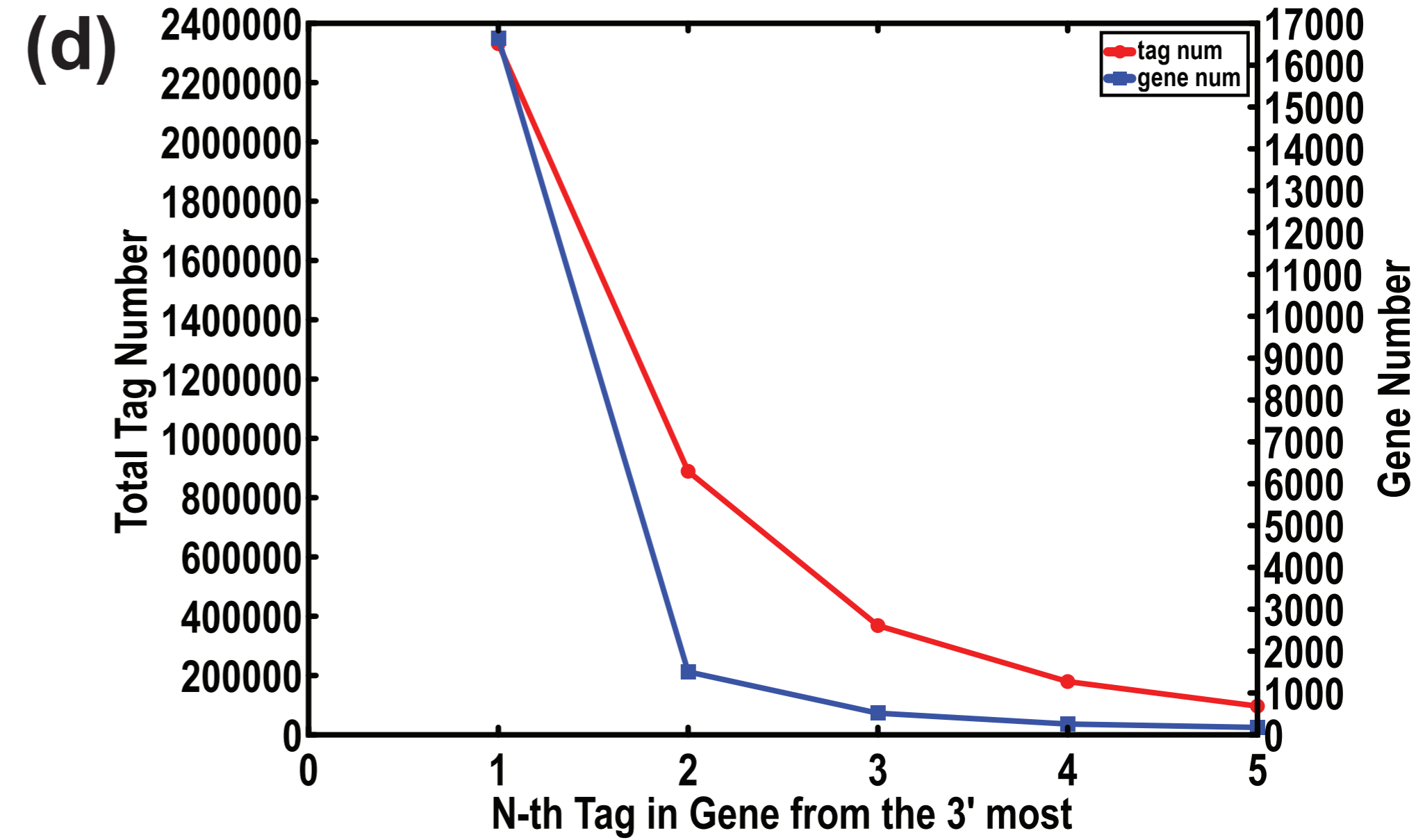

Supplement: Additional file 15 — Distribution of DGE tags across CATG sites within genes and gene numbers supported by DGE tags from different CATG sites. (a) japonica. (b) indica. (c) O. rufipogon. (d) O. nivara. For each panel, x-axis indicates CATG site positions from 3’-end of genes, while y-axis in left indicates the corresponding numbers of total tags mapped to different CATG positions and y-axis in right indicates the corresponding gene numbers supported by DGE tags mapped to different CATG sites. [file 1471-2164-13-300-S15.pdf]
